# Normative values of resting heart rate variability in young male contact sport athletes: Reference values for the assessment and treatment of concussion

**DOI:** 10.3389/fspor.2022.730401

**Published:** 2023-01-09

**Authors:** Hatem Ziadia, Idriss Sassi, François Trudeau, Philippe Fait

**Affiliations:** ^1^ Department of Anatomy, Université du Québec à Trois-Rivières, Trois-Rivières, QC, Canada; ^2^ Exercise Physiology Laboratory, Université du Québec à Trois-Rivières, Trois-Rivières, QC, Canada; ^3^ Department of Psychology, Université du Québec à Trois-Rivières, Trois-Rivières, QC, Canada; ^4^ Department of Human Kinetics, Université du Québec à Trois-Rivières, Trois-Rivières, QC, Canada; ^5^ Research Group on Neuromusculoskeletal Conditions (GRAN), Trois-rivieres, QC, Canada; ^6^ Centre for Research in Neuropsychology and Cognition (CERNEC), Montreal, QC, Canada; ^7^ Cortex Concussion Clinic, Quebec City, QC, Canada

**Keywords:** heart rate variability, normative values, heart rate correction, concussion, sports-related concussion, diagnosis, management, treatment biomarkers.

## Abstract

**Objective:**

The objective of this study was to identify the main determinants of heart rate variability (HRV) in male athletes aged 14 to 21 years who practice competitive contact sports and to integrate these determinants with the aim of defining normative values of short-term HRV in the time and frequency domains.

**Methods:**

Participants (*n* = 369) were aged 14 to 21 years and included 221 football players and 148 ice hockey players. HRV was measured for 5 min at rest, and standard HRV parameters in the time and frequency domains were calculated. Heart rate (HR), age, body mass index (BMI), number of sports weekly practices (WSP) and concussion history (mTBI) were considered determinants potentially able to influence HRV.

**Results:**

Multiple regression analysis revealed that HR was the primary determinant of standard HRV parameters. The models accounted for 13% to 55% of the total variance of HRV and the contribution of HR to this model was the strongest (*β* ranged from −0.34 to −0.75). HR was the only determinant that significantly contributes to all HRV parameters. To counteract this dependence, we calculated HRV corrected by the mean RR interval (RRm). Such corrections do not remove any physiological differences in HRV; they simply remove the mathematical bias. HRV parameters were therefore normalized, and their normative limits were developed relative to the mean heart rate. After correction, the correlation coefficients between HR and all corrected HRV parameters were not statistically significant and ranged from −0.001 to 0.045 (*p* > 0.40 for all). The automatically corrected HRV calculator, which recalculates standard HRV parameters and converts them into corrected parameters in addition to determining whether a given value is within normal limits, facilitates clinical interpretation.

**Conclusion:**

This study provides for the first time corrected normative values of short-term and resting state HRV parameters in competitive contact sport athletes aged 14 to 21 years. These values were developed independently of the major determinants of HRV. The baseline values for HRV parameters given here could be used in clinical practice when assessing and monitoring cerebral concussions. They may assist in decision making for a safe return to play.

## Introduction

A healthy heart is subject to complex and constant variations of its electrical rhythm. These oscillations allow the heart to rapidly adapt to environmental and psychological challenges to help achieve body homeostasis and optimal performance. Those cardiac oscillations, better known as HRV, are the fluctuation in the time intervals between successive heartbeats (the variation in cardiac cycle lengths, RR intervals) (1, 2). The HRV measurement is considered a standard, reliable and noninvasive tool that provides a quantitative assessment of autonomic control of cardiovascular function and offers insight into the balance of influences of the sympathetic (SNS) and parasympathetic nervous systems (PNS) (2, 3). Indeed, although our heart is an organ capable of functioning and reacting independently of neural control systems through the sinoatrial node, the genesis of cardiac automatism, its activities are strongly influenced by the functions of the autonomic nervous system (ANS) with its sympathetic and parasympathetic branches (4). These influences are known to be antagonistic. On one hand, sympathetic activity is primarily related to preparing the body for response in demanding or alarming situations, commonly referred to as the “fight or flight” response (5). On the other hand, parasympathetic activity is generally identified with the “rest and digest” response (5). Thus, the role of the sympathetic branch is to increase the chronotropic (heart rate) and inotropic (force of contraction) responses, and cardiac output therefore becomes greater in order to accommodate emergencies or exercise. In contrast to the sympathetic system, the parasympathetic branch works in more restful situations and slows down the effects of sympathetic activity with the consequence of restoring and maintaining a balanced state (1). The relationship between the sympathetic and parasympathetic branches is more complex and should not be described as a zero-sum system. Increased PNS activity may be associated with decreased, increased or no change in SNS activity (6, 7). However, HRV can be viewed as a mirror of the interactions between the SNS and PNS. The clinical utility of HRV as an indicator of SNS activity has been explored in numerous health studies subjects including diabetes (8, 9), hypertension (10, 11), cardiovascular pathophysiology (12, 13) and physiology (14) as well as in the assessment and management of psychological stress (15), psychopathology and neurocognitive disorders (16). In recent years, links have been made between HRV and traumatic brain injury (TBI) (17–21), mostly in research involving populations with moderate to severe TBI. In these studies, HRV was used to provide clues to the dysregulation of the ANS following trauma and monitor the evolution of autonomic parameters during post-traumatic recovery. The degree of alterations in post-TBI HRV parameters has been associated with trauma severity, intracranial pressure, functional outcomes and survival in both adults and children (21–29). The effectiveness of using HRV in the assessment and monitoring of TBI rehabilitation has encouraged other investigators to explore its effectiveness in the assessment of concussions (CC) (30–38).

At the 5th international conference on concussion in sport (39), guidelines were proposed emphasizing the need for diagnostic biomarkers such as advanced neuroimaging, fluid biomarkers and genetic testing. HRV could be a promising physiological non-invasive biomarker to help consolidate the multidimensional approach needed in the management of CC. Indeed, although CC is being at the “mild” end of the continuum of severity for craniocerebral injury, it has been described as a complex health problem that causes neurological, psychological and cognitive disorders that may manifest as symptoms such as headaches (40), difficulty concentrating, organizational problems, sleep problems (41–43), slowed information processing (44) and psychological problems including depression, anxiety and frustration (45, 46). While these symptoms are often transient (10–14 days), their resolution may not always coincide with physiological recovery, resulting in a potential window of brain vulnerability and risk of exposure to secondary injury (47, 48). In several studies, this vulnerability is reflected by a lower HRV attributed to impaired autonomic modulation as a likely contribution (33–38). Thus, HRV could be a promising clinical tool and an effective biomarker in the assessment and management of CC. However, in terms of clinical use, normative data must be established that consider the specifics of the study population and the methodological factors that may influence HRV interpretation. These factors include age (49), sex (50), physical activity level (51, 52), body mass index (BMI) (53, 54), breathing (55), the position of the individual during measurement (56), time of measurement (57), measurement times (1), sleep (58, 59), and stress level (60). It is therefore important to try to control for these factors in order to minimize biases that may complicate the interpretation of results. To date, no studies have reported specific normative reference values for athletic populations in contact sports such as football and ice hockey, which are characterized by a higher risk of CC. Indeed, in Canada, ice hockey is among the sports with the highest number of CCs in children and young people aged 5–19 years, accounting for up to 44% of all injuries that occur during play (61). In Ontario and Alberta, the rate of ice hockey CCs recorded in the emergency department in 2017–18 increased significantly in boys aged 10 to 14 years and reached 203.7 per 100,000 people (62). Ice hockey remains the category responsible for the majority of CCs associated with sports and recreation among boys aged 15–19 years (150.7 per 100,000 persons), followed closely by football (119.2 per 100,000 persons) (62). Marar et al. (63) report that out of 20 sports evaluated during 2008–2010, 47.1% of CCs were recorded in football. On the other hand, some athletes may have experienced CC without necessarily realizing it. In fact, 70% of football players reported experiencing symptoms of CC in a single season without being diagnosed with CC (64).

Despite significant advances in the assessment of CCs, clinicians face considerable challenges when using HRV to assess and treat this condition, particularly when the study population has specific characteristics. These features limit the effectiveness of using general normative population values when assessing and treating contact sport athletes. Thus, there is a concern with establishing normative values for HRV parameters specific to this particular population, values that take into account said population's anthropometric characteristics and follow standardized methodological guidelines that minimize the risk of influencing HRV interpretation.

The objectives of this study were (1) to identify the main determinants of HRV in athletes from competitive contact sports aged 14–21 years, and (2) to integrate these determinants in order to define normative values of short-term HRV in the time and frequency domain.

## Materials and methods

### Study population

The study group consisted of 464 competitive athletes from the Mauricie Quebec Student Sport Network (*Réseau Sport Étudiant Québec-Mauricie*). Data were collected at the start of the 2018–19 and 2019–20 competitive seasons. Male football players (*n* = 286) and male ice hockey players (*n* = 178) were evaluated. The Université du Québec à Trois-Rivières Human Research Ethics Board approved the research protocol, and all participants signed a written consent prior to participation (CER-16-230-07.16). To be eligible for this study, a player had to meet the following criteria: be a competitive football or ice hockey athlete; be aged 14 to 21 years; have suffered no CC during the six months prior to enrollment; with no moderate or severe TBIs and/or skull fracture; have no medical contraindication regarding physical exertion (Physical Activity Readiness Questionnaire, (PAR-Q) (65); and have no diseases and/or was taking no regular medications that affect the cardiopulmonary system and/or interfere with the autonomic nervous system. 95 participants (65 football players and 30 ice hockey players) were excluded from the analysis because of failure to comply with the methodological restrictions required by our experimental protocol. Data from 369 participants (221 football players and 148 ice hockey players) were included in the final analysis.

### Procedure

Two days before the assessment, participants received an email explaining the restrictions to observe in keeping with our experimental protocol. Regarding the time prior to evaluation, the restrictions prohibited physical exercise for 48 h (66, 67); alcohol for 24 h (68, 69); smoking for 12 h (70, 71); caffeine or energy drinks for 6 h (72, 73); and heavy meals for 4 h (74, 75).

All measurements were made in the afternoon between 1 and 6 pm in a classroom at the sports team's educational facility (57, 76). Environmental conditions were maintained at 20.2 ± 0.8°C temperature, 24.6 ± 4% humidity, and 100.9 ± 0.7 kPa pressure (Cole-Parmer Digital Barometer, model RS-232) (77). Participants’ anthropometric data were measured (body mass and height) and computed (body mass index). Before starting the HRV measurement, participants were instructed to empty their bladders in the toilet to avoid bladder distention (78–80) and were instructed not to drink more water until the measurements were completed (81–83). Each participant signed the consent form and completed a recruitment questionnaire. The questionnaire was used to determine the participants’ profile, i.e., history of CCs, frequency of weekly training (84–86), quality and number of hours of sleep the night before the measurements (58, 59), and whether the participant was under medical treatment or suffered from chronic migraines or attention deficit disorder with or without hyperactivity (ADHD). After the completed questionnaires were reviewed, the data from 95 participants were rejected for non-compliance with control items (65 football players and 30 ice hockey players). Most of these players had not abstained from physical activity during the 48 h preceding the tests (64.2%) (66, 67), had consumed caffeine during the 6 h preceding the evaluation (14.7%) (72, 73), had eaten a meal during the 4 h preceding the evaluation (10.5%) (74, 75), had taken medication for a health problem (8.4%) and had consumed alcohol during the 24 h preceding the evaluation (2.1%) (68, 69). After completing the questionnaires, each participant was fitted with a chest heart rate belt (POLAR Team2 Pro transmitter, Polar Electro Oy, Kempele, Finland). A check was made to ensure that the electrode areas of the belt were properly wetted and the belt was properly fitted and felt comfortable. The installation of the belt at the beginning of the protocol allowed the participant to become familiar with the equipment (73, 87). This first step also enabled the stabilization of physiological variables in the same body position that would be used for the HRV measurement (73, 88, 89).

### Recording and analysis of heart rate variability

First, participants were asked to count their spontaneous breaths for one minute to measure respiratory rate (RR) and record it on the recruitment questionnaire (55, 90, 91). To eliminate the risk of interference with HRV data collection, participants removed clothing that could generate small static discharges and performed the data collection shirtless. For the same reasons, all participants’ electronic equipment was turned off (phones, watches, computers, etc.) (73). To standardize the HRV measurements, each participant was seated in a rigid chair with feet flat and knees bent at 90°, back straight and resting firmly on the backrest, arm resting on the desk at 90°, and eyes closed (56, 92). All measurements were made in waves of 20 participants. Indeed, to measure HRV, we used the POLAR Team2 Pro device, Polar Electro Oy, Kempele, Finland, which can collect data from 80 participants at the same time (93–95). This tool digitizes the electrocardiogram at a resolution of 1,000 Hz and determines the RR interval to the nearest millisecond. These technical characteristics are above the minimum required by expert consensus for HRV analysis (1, 93–95). During the 7 min of measurement, transmitter is attached to the Team2 transmitter strap, and the strap is secured around player's chest. The transmitter of each player tested records HR and HRV data and transmits it to the PC using Bluetooth. A visual check of the signal was made in real-time. A repeat of the measurements was made for participants who gave an unstable signal. From the 7 min of recording, only the last 5 min were retained for analysis, the first two minutes of recording allowing the attainment of a steady state for physiological parameters such as HR, RF and HRV (96, 97). No attempt was made to control the participants' respiratory rate or tidal breathing volume (98). Time-and frequency-domain HRV analyses were performed using Kubios HRV Standard 3.3.0 software (University of Eastern Finland, Kuopio, Finland) (99, 100). This software supports several input data formats for electrocardiogram (ECG) and RR beat-to-beat interval recordings. It calculates all time-and- frequency-domain HRV parameters and even several non-linear parameters. The software includes an adaptive QRS detection algorithm and analysis sample selection and artifact correction tools. Thus, RR interval data were entered into Kubios, the appropriate correction threshold was selected by visual inspection of the tachogram to ensure the correction removed artifacts but did not distort the normal RR intervals. When the correction was applied, detected artifact beats were automatically replaced by cubic spline interpolation (99). Less than 1% of RR intervals were corrected for our participants. The results of the RR interval-time-domain component analysis were expressed as the standard deviation of RR intervals (SDNN), square root of the mean of the sum of squared differences between adjacent RR intervals (RMSSD), percentage of RR intervals >50 ms from the previous (pNN50). Frequency-domain analyses were performed by fast Fourier transform (FFT) and autoregressive (AR) modelling. The very low frequency (VLF, 0–0.04 Hz), the low frequency (LF, 0.04–0.15 Hz), the high frequency (HF, 0.15–0.40 Hz) and the total power according to its two forms, i.e., TP 1 (VLF + LF + HF, 0–0.5 Hz) and TP 2 (LF + HF, 0.04–0.50 Hz)] were expressed in absolute values (ms2). The LF / HF ratio was also calculated. The normalized (nu) powers of the LF and HF bands (nLF and nHF) were calculated as follows: nLF (the normalized LF power) = LF/[total power (ms2)—VLF (ms2)] and nHF (the normalized HF power) = HF/[total power (ms2)—VLF (ms2)], respectively (1).

### Normalization of heart rate variability to the average heart rate

HRV is significantly associated with mean HR through the influence of physiological phenomena and mathematical constraints. The physiological phenomenon arises from the activity of the autonomic nervous system and its control of the heartbeat (1). The mathematical determinant is caused by the inverted nonlinear relationship between the RR interval and HR (101). Thus, when analyzing RR intervals, the same changes in HR cause much higher fluctuations in RR intervals for slow average HR compared to fast average HR (101). This phenomenon mathematically amplifies the effect of ANS influence on HRV and can cause an artificial difference in HRV parameters to appear in patients with different mean HR only through mathematical constraint (101). This makes the standard HRV analysis mathematically biased, especially if patients differ in terms of their mean HR (101–103). However, this bias can be excluded by a simple mathematical modification of normalizing RR fluctuations to the mean. This is done by multiplying or dividing the standard HRV indices by the corresponding average RR intervals (101–103). Thus, if HRV parameters are negatively related to HR, they should be divided by the appropriate mean RR power to become independent of HR. Conversely, if HRV parameters are positively related to HR, they should be multiplied by the appropriate average RR power. This technique has been shown to be effective for eliminating the mathematical dependence of HRV on HR and has allowed for exploration of a true clinical value of HR and its variability (104).

### Statistical analysis

All statistical analyses were performed using IBM SPSS Statistics v. 27. The corrected HRV normative value calculator was created with Microsoft Excel 365.

With reference to to previous studies aimed at determining normative values for HRV, heart rate (HR), age, body mass index, number of sports practices per week and CC history were considered determinants that could potentially influence HRV (51, 105–110).

The normality of the distributions of each HRV parameter and of the potential determinants of HRV parameters was tested using the one-sample Kolmogorov-Smirnov test before and after log transformation. Variables that did not show a normal distribution were natural log-transformed because of their positive skewness. Conclusions about the normality of the variables were reinforced by checking the values of z(g1) for skewness and z(g2) kurtosis (±3), as well as by visual interpretation of the normal Q-Q plot and the histogram graph. Normally distributed parameter values are presented as mean ± standard deviation, whereas abnormally distributed values are presented as median and 25th—75th percentiles. The significant level is *p* ≤ 0.05.

Because some of the HRV parameters and predictors did not pass the normality test even after log transformation, nonparametric tests were used. Thus, to establish the relationship between the different HRV predictors, we used the Spearman correlation coefficient (rS). To test for significant differences between variables, we used the Wilcoxon-Mann-Whitney test. For comparisons of more than 2 groups, we used the Kruskal-Wallis (K-W) test. Standard multiple regression analysis was performed with all standard and adjusted HRV variables to determine significant predictors of HRV. To avoid collinearity, we checked the correlation between all predictors, validating the absence of collinearity in each regression with Python variance inflation factor values (VIF < 10). The effect size of each predictor and the entire regression model for each variable is calculated separately with Cohen's f2 index. According to Cohen's scale, f2 ≥ 0.02, f2 ≥ 0.15 and f2 ≥ 0.35 represent a weak, medium and strong effect size, respectively.

## Results

A total of 369 participants (221 football players and 148 ice hockey players) aged 14–21 years were included in the analysis. Group characteristics were presented as median, 25th to 75th percentiles as follows: age 17.8; (16.1–19.1 years); body mass, 77.1 kg (68–88.5 kg), height; 1.78 m (1.73–183 m); BMI, 24.3 kg/m^2^ (21.9–26.8 kg/m^2^); history of CCs, 0 (0–1); and weekly sports practices, 5 (4–6). Mean HR was 71.4 ± 10.1 bpm and mean RF was 13.9 ± 2.9 rpm.

The Kolmogorov-Smirnov test was used to assess the normality of the data distribution. Of the variables initially chosen as potential determinants of standard HRV parameters in contact sport athletes (HR, heart rate, BMI, body mass index, n.mTBI, number of mild traumatic brain injury and WSP, weekly sport practice), only HR showed a normal distribution. Age, BMI, n. mTBI, and WSP were not normally distributed. Correlation analysis between these predictors revealed that all variables had a significant relationship with each other except for n.mTBI, which was not statistically significantly correlated with HR and BMI (Table 1). In these analyses, the strongest correlation was between age and BMI with *R* = 0.45; *p* < 0.001. All predictors did not show a collinearity effect between them, which was confirmed by the collinearity test where the values were not just below 10 but even <1.33; 1.03>. A VIF under 1.5 indicates the total absence of collinearity. The strong significant value obtained between the protector (Table 1) was due to the natural relationship between the variables and the sensibility of the test with the number of participants (*n* = 369). On the other hand, correlation analysis between these predictors and HRV showed that all HRV parameters correlated significantly with HR. Next, BMI correlated with 14 out of 19 HRV parameters. The n.mTIB showed a significant correlation only with two parameters (LF and VLF). Although WSP and age did not show a significant correlation with HRV, they were mentioned based on the respective closeness of their correlation coefficient with HRV parameters (Table 2).

**Table 1:** Association between variables selected as potential independent predictors of standard HRV parameters.

Parameter	Mean HR	Age	BMI	n.mTBI	WSP
Mean HR (min^−1^)	1	** **	** **	** **	** **
Age (years)	−0.21***	1	** **	** **	** **
BMI (kg/m^2^)	−0.14***	0.45***	1	** **	** **
n. mTBI	−0.07	0.14**	0.1	1	** **
WSP	−0.2***	0.36***	0.27***	0.15**	1

Mean HR, mean heart rate; BMI, body mass index; n.mTBI, number of mild traumatic brain injury; and WSP, weekly sport practice.

**p < 0.01; ***p < 0.001.

**Table 2:** Correlations of standard time and frequency-domain HRV parameters with mean heart rate, age, body mass index, number of mild traumatic brain injury and weekly sport practice.

Standard HRV Parameter	Mean HR (bpm)		Age (months)		BMI (kg/m^2^)		n. mTBI		WSP	
	R	*P*	R	*P*	R	*P*	R	*P*	R	*P*
SDNN (ms)	−0.48	<0.001	0.01	0.79	0.09	0.07	0.09	0.08	0.07	0.17
RMSSD (ms)	−0.64	<0.001	0.01	0.83	0.14	<0.01	0.05	0.32	0.07	0.18
pNN50 (%)	−0.67	<0.001	0.02	0.67	0.15	<0.001	0.06	0.27	0.07	0.18
FFT VLF (ms^2^)	−0.32	<0.001	0.07	0.16	0.09	0.10	0.08	0.13	0.08	0.14
FFT LF (ms^2^)	−0.30	<0.001	0.04	0.45	−0.01	0.85	0.09	0.09	0.06	0.24
FFT HF (ms^2^)	−0.70	<0.001	0.04	0.44	0.14	<0.01	0.04	0.44	0.07	0.19
FFT TP1 (ms^2^)	−0.61	<0.001	0.05	0.30	0.10	<0.05	0.07	0.21	0.08	0.10
FFT TP2 (ms^2^)	−0.61	<0.001	0.05	0.33	0.10	<0.05	0.07	0.20	0.08	0.12
FFT LF/HF	0.56	<0.001	0.01	0.99	−0.20	<0.001	0.01	0.86	−0.01	0.78
FFT nLF (nu)	0.56	<0.001	0.01	0.97	−0.20	<0.001	0.01	0.87	−0.01	0.78
FFT nHF (nu)	−0.56	<0.001	0.01	0.98	0.20	<0.001	−0.01	0.84	0.01	0.80
AR VLF (ms^2^)	−0.47	<0.001	0.07	0.18	0.07	0.17	0.10	<0.05	0.07	0.18
AR LF (ms^2^)	−0.25	<0.001	0.05	0.31	0.03	0.51	0.11	<0.04	0.10	0.06
AR HF (ms^2^)	−0.72	<0.001	0.03	0.55	0.15	<0.01	0.04	0.45	0.06	0.22
AR TP1 (ms^2^)	−0.63	<0.001	0.06	0.24	0.13	<0.05	0.08	0.15	0.10	0.06
AR TP2 (ms^2^)	−0.62	<0.001	0.06	0.27	0.13	<0.05	0.07	0.16	0.10	0.07
AR LF/HF	0.57	<0.001	0.02	0.67	−0.15	<0.01	0.05	0.36	0.03	0.56
AR nLF (nu)	0.57	<0.001	0.02	0.67	−0.14	<0.01	0.05	0.37	0.03	0.55
AR nHF (nu)	−0.57	<0.001	−0.02	0.67	0.15	<0.01	−0.05	0.36	−0.03	0.56

Mean HR, mean heart rate, BMI, body mass index, n.mTBI, number of mild traumatic brain injury and WSP, weekly sport practice. FFT denotes that a given HRV parameter was calculated by using Fast Fourier Transform, while AR indicates that the HRV spectrum was calculated with autoregressive method. R, Spearman's rank correlation coefficient. nu, normal units.

The results of the multiple regression analysis for time-and-frequency-domain HRV parameters calculated with FFT, including HR, age, BMI, n.mTBI and WSP are presented in Table 3. The results of this analysis show that HR was the strongest determinant for all HRV parameters for the entire group as well as for all age subgroups (Table 3 and the table in Supplementary Appendix SA1 and SA3–10). The models accounted for 13%–55% of the total variance in HRV. The contribution of HR to this model was significant (*β* ranged from −0.34 to −0.75). The group was divided into four subgroups by age: (1) 14 ≤ age <16 years; (2) 16 ≤ age <18 years; and (3) 18 ≤ age <20 years and (4) 20 ≤ age <22 years. The number of football (F) and ice hockey (H) players in consecutive age subgroups was 89 (F:44/H:45), 113 (69 / 44), 120 (84/36), and 47 (24/23). The four age subgroups show a similarity in standard HRV parameters, where the Kruskall-Wallis test was higher than 0.05 for all.

**Table 3:** Determinants of standard time and frequency-domain HRV parameters calculated with fast-Fourier transform.

Standard HRV parameter	Determinant	Parameters of multiple regression analysis					
		*β*	*P*	Partial correlation	Multiple R^2^	F-test	*P*
SDNN (ln)	Mean HR	−0.52	<0.001	−0.51	0.27	26.75	<0.001
	Age (ln)	−0.14	<0.01	−0.12			
	BMI (ln)	0.06	0.27	0.05			
	n.mTIB	0.06	0.19	0.06			
	WSP (ln)	−0.02	0.76	−0.01			
RMSSD (ln)	Mean HR	−0.71	<0.001	−0.68	0.48	67.51	<0.001
	Age (ln)	−0.17	<0.001	−0.15			
	BMI (ln)	0.10	<0.05	0.09			
	n.mTIB	0.04	0.34	0.04			
	WSP (ln)	−0.06	0.17	−0.05			
pNN50 (ln)	Mean HR	−0.72	<0.001	−0.69	0.50	71.39	<0.001
	Age (ln)	−0.18	<0.001	−0.15			
	BMI (ln)	0.12	<0.01	0.10			
	n.mTIB	0.05	0.16	0.05			
	WSP (ln)	−0.09	<0.05	−0.08			
VLF (ln)	Mean HR	−0.35	<0.001	−0.34	0.13	11.15	<0.001
	Age (ln)	0.00	0.99	0.00			
	BMI (ln)	0.01	0.84	0.01			
	n.mTIB	0.05	0.33	0.05			
	WSP (ln)	0.02	0.75	0.02			
LF (ln)	Mean HR	−0.34	<0.001	−0.33	0.13	10.41	<0.001
	Age (ln)	−0.02	0.68	−0.02			
	BMI (ln)	−0.10	0.06	−0.09			
	n.mTIB	0.06	0.24	0.06			
	WSP (ln)	0.02	0.75	0.02			
HF (ln)	Mean HR	−0.75	<0.001	−0.73	0.55	88.10	<0.001
	Age (ln)	−0.16	<0.001	−0.13			
	BMI (ln)	0.12	<0.001	0.11			
	n.mTIB	0.02	0.57	0.02			
	WSP (ln)	−0.08	<0.05	−0.07			
TP1 (ln)	Mean HR	−0.66	<0.001	−0.64	0.42	51.75	<0.001
	Age (ln)	−0.12	<0.01	−0.10			
	BMI (ln)	0.04	0.33	0.04			
	n.mTIB	0.04	0.35	0.04			
	WSP (ln)	−0.04	0.37	−0.04			
TP2 (ln)	Mean HR	−0.66	<0.001	−0.64	0.42	51.75	<0.001
	Age (ln)	−0.12	<0.01	−0.10			
	BMI (ln)	0.04	0.34	0.04			
	n.mTIB	0.04	0.34	0.04			
	WSP (ln)	−0.04	0.34	−0.04			
LF/HF (ln)	Mean HR	0.62	<0.001	0.60	0.41	50.35	<0.001
	Age (ln)	0.17	<0.001	0.15			
	BMI (ln)	−0.25	<0.001	−0.22			
	n.mTIB	0.03	0.45	0.03			
	WSP (ln)	0.11	<0.01	0.11			
nLF	Mean HR	0.61	<0.001	0.59	0.41	49.45	<0.001
	Age (ln)	0.18	<0.001	0.15			
	BMI (ln)	−0.25	<0.001	−0.22			
	n.mTIB	0.02	0.55	0.02			
	WSP (ln)	0.12	<0.01	0.11			
nHF	Mean HR	−0.61	<0.001	−0.59	0.40	48.97	<0.001
	Age (ln)	−0.18	<0.001	−0.15			
	BMI (ln)	0.25	<0.001	0.22			
	n.mTIB	−0.03	0.53	−0.03			
	WSP (ln)	−0.12	<0.01	−0.11			

Ln, natural logarithm.

The standard values of HRV parameters in the time-and-frequency domain of the general group and its four subgroups are presented in Table 4 as median, 5th to 95th percentiles. Indeed, because HRV is significantly associated with HR, and HR is the strongest determinant for all its standard parameters, they were classified into four subgroups according to the following HR quartiles: 1st quartile (Q1: <25%), 48.9–63.7 bpm; 2nd quartile (Q2: 25%–50%), >63.7–71.4 bpm; 3rd quartile (Q3: 50%–75%), >71.4–78.4 bpm; and 4th quartile (Q4: >75%), >78.4–107.7 bpm. The number of participants in each subgroup ranged from 92 to 93. Comparison of these groups revealed there was a significant difference between all HRV parameters based on the four HR quartiles. The Kruskal-Wallis rank test ranged from 32.75 to 160.78 with *p* < 0.001 for all comparisons.

**Table 4:** Normative standard time and frequency-domain HRV parameters values for overall study group and according to heart rate quartiles are presented as median, 5th–95th percentiles.

Standard HRV Parameter	Overall study group (n: 369)	Quartiles according to HR			
		Q1 (n: 93)	Q2 (n: 92)	Q3 (n: 92)	Q4 (n: 92)
SDNN (ms)	53 (29–92)	62 (40–107)	56 (32–92)	49 (29–83)	43 (24–71)
RMSSD (ms)	37 (17–77)	53 (32–90)	43 (24–68)	33 (20–57)	26 (13–47)
pNN50 (%)	12 (1–47)	27 (9–57)	16 (3–41)	9 (2–28)	5 (0–16)
FFT VLF (ms^2^)	88 (21–369)	123 (41–601)	108 (29–381)	83 (20–279)	66 (13–236)
FFT LF (ms^2^)	1,479 (426–4,415)	1,834 (632–5,786)	1,746 (612–4,408)	1,368 (393–4,349)	1,088 (280–3,095)
FFT HF (ms^2^)	1,818 (307–7,851)	4,062 (1,528–11,718)	2,335 (843–5,896)	1,357 (382–3,913)	818 (159–2,737)
FFT TP1 (ms^2^)	3,733 (913–11,217)	6,353 (2,368–15,699)	4,387 (1,629–10,001)	2,930 (980–8,145)	2,058 (659–5,559)
FFT TP2 (ms^2^)	3,525 (868–10,993)	6,199 (2,266–15,567)	4,271 (1,575–9,728)	2,892 (899–8,019)	1,957 (643–5,363)
FFT LF/HF	0 (0–2)	0.46 (0.14–1.48)	0.73 (0.28–1.61)	1.32 (0.27–2.61)	1.43 (0.4–4.31)
FFT nLF (nu)	47 (17–73)	31 (12–59)	42 (22–61)	56 (21–71)	58 (28–81)
FFT nHF (nu)	52 (26–82)	68 (40–86)	57 (38–77)	42 (27–78)	40 (18–71)
AR VLF (ms^2^)	268 (81–647)	372 (123–791)	296 (113–690)	236 (87–599)	165 (55–461)
AR LF (ms^2^)	1,302 (345–4,476)	1,584 (570–6,021)	1,482 (406–4,476)	1,105 (277–4,430)	1,016 (225–2,957)
AR HF (ms^2^)	1,861 (316–8,168)	4,237 (1,511–11,578)	2,473 (875–6,448)	1,493 (463–3,502)	765 (171–2,701)
AR TP1 (ms^2^)	3,768 (1,030–12,466)	6,304 (2,776–15,790)	4,358 (1,517–10,472)	3,048 (1,009–8,068)	2,116 (627–5,749)
AR TP2 (ms^2^)	3,414 (884–11,925)	5,861 (2,570–15,117)	4,097 (1,340–9,844)	2,710 (897–7,633)	1,971 (561–5,392)
AR LF/HF	0 (0–2)	0.4 (0.11–1.29)	0.64 (0.22–1.75)	0.98 (0.23–2.03)	1.29 (0.26–3.7)
AR nLF (nu)	43 (13–71)	28 (10–56)	38 (18–63)	49 (18–66)	56 (20–78)
AR nHF (nu)	56 (27–85)	70 (43–89)	60 (36–81)	50 (32–80)	43 (21–79)

FFT denotes that a given HRV parameter was calculated by using Fast Fourier Transform, while AR indicates that the HRV spectrum was calculated with autoregressive method. HR quartiles for overall study group: (Q1), 48.9–63.7 bpm; (Q2), >63.7–71.4 bpm; (Q3), >71.4–78.4 bpm; (Q4), >78.4–107.7 bpm. nu, normal units.

Multiple regression models involving potential variables capable of influencing HRV parameters (HR, age, BMI, n. mTBI, and WSP) revealed that HR was the strongest determinant for influencing HRV parameters. To counteract this dependence, we calculated HRV corrected by the mean RR interval (RRm). This correction method consists of dividing or multiplying the HRV parameters by RRm power (104). Such corrections do not remove any physiological differences in HRV between heart rates and different mean HRs; they simply remove the mathematical bias (101, 111). Thus, the standard time-domain HRV parameters SDNN, RMSSD, and pNN50 lost their dependence on HR after being divided by RRm to the power of 1.2; 2 and 4.35, respectively. In the frequency domain analysis, the VLF, LF, HF, TP1, TP2, and nHF parameters obtained by FFT lost their CF dependencies after being divided by RRm at the powers 2.1; 1.55; 4.5; 3.1; 3.1; and 1.33, respectively. The remaining standard HRV parameters obtained by FFT (LF / HF and nLF) lost their CF dependencies after being multiplied by RRm at power 3.1 and 1.6, respectively. For the frequency domain analysis obtained by AR, the VLF, LF, HF, TP1, TP2, and nHF parameters were divided by RRm at powers 2; 1.45; 4.65; 3.15; 3.25; and 1.4, respectively. LF / HF and nLF were multiplied by RRm to the power 3.4 and 1.85, respectively (113). With these simple mathematical corrections, we were able to obtain new normative HRV values independent of mean HR that were termed corrected HRV (HRV_corr_). After correction, the correlation coefficients between HR and all corrected HRV parameters were not statistically significant and ranged from −0.001 to 0.045 (*p* > 0.40 for all).

After correction, multiple regression analysis involving age, BMI, n. mTBI and WSP revealed that age was the strongest determinant that could influence HRV_corr_ parameters in the time and frequency domain calculated with FFT and AR (Table 5 and Supplementary Appendix SA2 Table). However, the calculated patterns accounted for only 1%–13% of the total variance of the HRV_corr_ indices. The contribution of age to this model was quite small (*β* ranged from −0.001 to −0.23). In addition, the effect size for the combined model as well as for the individual variables (age, BMI, n. mTBI and WSP) was small (f2 ≤ 0.01 for all). CC history was the only variable that showed no influence on HRV parameters. These results reflect the exclusion of any dependence of HRV_corr_ on other model parameters, which allowed us to establish corrected normative values of HRV parameters and their normal limits (Table 6). The normative values in Table 6 are presented in their scientific forms to eliminate the long number of digits after the decimal point due to division by the high powers of mean RR.

**Table 5:** Determinants and Cohen's f2 indexes for corrected time- and frequency-domain HRV parameters calculated with fast-Fourier transform.

Standard HRV parameter	Determinant	Parameters of multiple regression analysis						Cohen's f^2^	
		β	*P*	Partial correlation	Multiple R^2^	F-test	*P*	Local	Combined
Corr-SDNN	Age (months)	−0.13	<0.05	−0.12	0.019	1.742	0.140	0.014	<0.001
	BMI	0.04	0.47	0.04				0.001	
	n.mTIB	0.08	0.14	0.08				0.006	
	WSP	−0.01	0.93	0.001				0.000	
Corr-RMSSD	Age (months)	−0.20	<0.001	−0.18	0.045	4.28	<0.01	0.032	<0.01
	BMI	0.10	0.07	0.09				0.009	
	n.mTIB	0.06	0.26	0.06				0.003	
	WSP	−0.06	0.26	−0.06				0.003	
Corr-pNN50	Age (months)	−0.19	<0.001	−0.16	0.047	4.53	<0.001	0.028	<0.01
	BMI	0.13	<0.05	0.12				0.014	
	n.mTIB	0.06	0.26	0.06				0.003	
	WSP	−0.09	0.10	−0.08				0.007	
Corr-VLF	Age (months)	0.01	0.90	0.01	0.005	0.43	0.787	0.000	<0.001
	BMI	0.02	0.75	0.02				0.000	
	n.mTIB	0.04	0.49	0.04				0.001	
	WSP	0.04	0.46	0.04				0.002	
Corr-LF	Age (months)	0.00	0.98	0.00	0.012	1.12	0.348	0.000	<0.001
	BMI	−0.08	0.15	−0.08				0.006	
	n.mTIB	0.07	0.16	0.07				0.005	
	WSP	0.03	0.60	0.03				0.001	
Corr-HF	Age (months)	−0.21	<0.001	−0.18	0.054	5.18	<0.001	0.035	<0.01
	BMI	0.16	<0.01	0.14				0.021	
	n.mTIB	0.04	0.46	0.04				0.001	
	WSP	−0.07	0.18	−0.07				0.005	
Corr-TP1	Age (months)	−0.12	<0.05	−0.10	0.015	1.42	0.228	0.011	<0.001
	BMI	0.04	0.49	0.04				0.001	
	n.mTIB	0.07	0.21	0.07				0.004	
	WSP	−0.02	0.73	−0.02				0.000	
Corr-TP2	Age (months)	−0.12	<0.05	−0.10	0.016	1.44	0.219	0.011	<0.001
	BMI	0.04	0.49	0.04				0.001	
	n.mTIB	0.07	0.21	0.07				0.004	
	WSP	−0.02	0.71	−0.02				0.000	
Corr-LF/HF	Age (months)	0.16	<0.001	0.14	0.080	7.94	<0.001	0.021	<0.01
	BMI	−0.25	<0.001	−0.23				0.054	
	n.mTIB	0.04	0.46	0.04				0.001	
	WSP	0.14	<0.01	0.13				0.017	
Corr-nLF	Age (months)	0.18	<0.001	0.15	0.085	8.43	<0.001	0.025	<0.01
	BMI	−0.27	<0.001	−0.25				0.066	
	n.mTIB	0.03	0.60	0.03				0.001	
	WSP	0.12	<0.05	0.11				0.012	
Corr-nHF	Age (months)	−0.21	<0.001	−0.18	0.117	12.02	<0.001	0.034	<0.05
	BMI	0.32	<0.001	0.29				0.092	
	n.mTIB	−0.03	0.61	−0.03				0.001	
	WSP	−0.15	<0.01	−0.13				0.018	

Corrected HRV rameters were calculated as follows: corr-SDNN, SDNN/mRR^1.2; corr-RMSSD, RMSSD/mRR^2.0; corrpNN50, pNN50/mRR^4.35; for FFT: corr-VLF, VLF/mRR^2.1; corr-LF, LF/mRR^1.55; corr-HF, HF/mRR^4.5; corr-TP1, TP1/mRR^3.1; corr-TP2, TP2/mRR^3.1; corr-LF/HF, LF/HF mRR^3.1; corr-nLF, nLF mRR^1.6; and corr-nHF, nHF/mRR^1.33. for AR: corr-VLF, VLF/mRR^2.0; corr-LF, LF/mRR^1.45; corr-HF, HF/mRR^4.65; corr-TP1, TP1/mRR^3.15; corr-TP2, TP2/mRR^3.25; corr-LF/HF, LF/HF mRR^3.4; corr-nLF, nLF mRR^1.85; and corr-nHF, nHF/mRR^1.4.

**Table 6:** Normative values for corrected HRV parameters for the whole study group.

Standard HRV Parameter	Normative values		
	Median	5th percentile	95th percentile
corr-SDNN (ms)	1.60 × 10^−2^	9.63 × 10^−3^	2.62 × 10^−2^
corr-RMSSD (ms)	5.25 × 10^−5^	3.20 × 10^−5^	8.69 × 10^−5^
corr-pNN50 (%)	2.28 × 10^−12^	4.21 × 10^−13^	5.97 × 10^−12^
FFT corr-VLF (ms^2^)	6.46 × 10^−5^	1.68 × 10^−5^	2.45 × 10^−4^
FFT corr-LF (ms^2^)	4.19 × 10^−2^	1.32 × 10^−2^	1.24 × 10^−1^
FFT corr-HF (ms^2^)	1.19 × 10^−10^	3.41 × 10^−11^	3.17 × 10^−10^
FFT corr-TP1 (ms^2^)	3.05 × 10^−6^	1.02 × 10^−6^	7.61 × 10^−6^
FFT corr-TP2 (ms^2^)	2.93E × 10^−6^	9.88 × 10^−7^	7.39 × 10^−6^
FFT corr-LF/HF	1.04 × 10^9^	3.34 × 10^8^	2.63 × 10^9^
FFT corr-nLF (nu)	2.20 × 10^6^	1.02 × 10^6^	3.33 × 10^6^
FFT corr-nHF (nu)	6.67 × 10^−3^	4.03 × 10^−3^	1.00 × 10^−2^
AR corr-VLF (ms^2^)	3.75 × 10^−4^	1.30 × 10^−4^	8.80 × 10^−4^
AR corr-LF (ms^2^)	7.27 × 10^−2^	2.05 × 10^−2^	2.30 × 10^−1^
AR corr-HF (ms^2^)	4.39 × 10^−11^	1.55 × 10^−11^	1.24 × 10^−10^
AR corr-TP1 (ms^2^)	2.17 × 10^−6^	7.68 × 10^−7^	5.39 × 10^−6^
AR corr-TP2 (ms^2^)	1.01 × 10^−6^	3.50 × 10^−7^	2.66 × 10^−6^
AR corr-LF/HF	6.88 × 10^9^	1.91 × 10^9^	1.73 × 10^10^
AR corr-nLF (nu)	1.09 × 10^7^	4.45 × 10^6^	1.72 × 10^7^
AR corr-nHF (nu)	4.40 × 10^−3^	2.65 × 10^−3^	6.60 × 10^−3^

FFT denotes that a given HRV parameter was calculated by using Fast Fourier Transform, while AR indicates that the HRV spectrum was calculated with autoregressive method. Corrected HRV parameters were calculated as follows: corr-SDNN, SDNN/mRR^1.2; corr-RMSSD, RMSSD/mRR^2.0; corrpNN50, pNN50/mRR^4.35; for FFT: corr-VLF, VLF/mRR^2.1; corr-LF, LF/mRR^1.55; corr-HF, HF/mRR^4.5; corr-TP1, TP1/mRR^3.1; corr-TP2, TP2/mRR^3.1; corr-LF/HF, LF/HF mRR^3.1; corr-nLF, nLF mRR^1.6; and corr-nHF, nHF/mRR^1.33. for AR: corr-VLF, VLF/mRR^2.0; corr-LF, LF/mRR^1.45; corr-HF, HF/mRR^4.65; corr-TP1, TP1/mRR^3.15; corr-TP2, TP2/mRR^3.25; corr-LF/HF, LF/HF mRR^3.4; corr-nLF, nLF mRR^1.85; and corr-nHF, nHF/mRR^1.4. nu, normal units.

## Discussion

The present study aimed to identify the main determinants of HRV in competitive contact sport athletes aged 14–21 years and integrate these determinants so as to define normative values of short-term HRV in the time-and-frequency domain. These normative values could be used in clinical practice as a reference during evaluation of CC and follow-up to rehabilitation. Indeed, HRV is commonly used as an index for assessing ANS function and its control on the myocardium (112). A high HRV promotes improved information transmission between the ANS and the myocardium, reflecting good behavioural adaptation and high cognitive flexibility. In contrast, low HRV reflects less interaction between these two components and does not reproduce essential information related to environmental changes (97, 113). Following CC, low resting HRVs have been reported during the acute recovery phase, which could persist beyond return to the subacute play phase (31, 34). Senthinathan et al. (34) in fact report disruptions in HRV (HFnu and LFnu) at rest, at 7 days after CC and even after the athletes returned to play, suggesting persistent disruptions in the ANS balance beyond symptom resolution. Purkayastha et al. (31) report that HRV (pNN50) was significantly lower at 3 days after CC in contact sport athletes compared to the healthy control group. The pNN50 value had become comparable to that of the control group by day 21 post-injury. These observations highlight the value of assessing HRV during the clinical management of CC and during validation of return to play. However, to be used clinically, normative data specific to the study group must be established that consider the key factors that can influence HRV, such as mean resting HR, age, BMI, physical activity level and CC history in addition to the techniques and methods used during assessment. The present study provides the first baseline values for corrected resting and short-term HRV parameters specific to competitive contact sport athletes aged 14–21 years.

Correlation analysis between factors that may influence HRV (mean resting HR, age, BMI, physical activity level and CC history) showed that all variables were significantly correlated with each other except for n.mTBI, which was nonsignificant with HR and BMI. The strongest correlation was between age and BMI (*R* = 0.45; *p* < 0.001). However, this relationship did not show a collinearity effect. Similar values were recorded in the study by Gąsior et al. (106), where a correlation between age and BMI was validated (*R* = 0.44; *p* < 0.001). In the latter study, however, BMI was considered redundant because of its collinearity with age, and age alone was included in the multivariate analysis. The purpose of the study by Gąsior et al. (106) was to validate normative values of HRVcorr parameters in 312 nonathletic, male and female, school-aged children (6–13 years). Although the number of participants in the latter study was comparable to ours, the collinearity results between age and BMI differed. We believe these differences are due to the specific characteristics of our participants, especially the football players who represented 60% of the general study population. Indeed, the anthropometric characteristics of football players, particularly BMI, differ according to the position of play (114), which may artificially influence the relationship between BMI and age. In our study group, the BMI of football players was higher than that of ice hockey players (median; 5th to 95th percentiles, respectively, 25.1; 18.8 to 37.4 kg/m^2^ and 22.9; 18.1 to 28.5 kg/m^2^). This difference was primarily influenced by a high BMI in 20% of the football players, which was greater than 28.5 kg/m^2^ (95th percentiles of the BMI of ice hockey players). These results support the need to focus on the anthropometric specificities of the study population when assessing HRV.

The results of multiple regression analysis of HRV parameters with HR, age, BMI, n.mTBI and WSP showed that HR was the strongest determinant for all HRV parameters for the general group (Table 3 and Supplementary Table SA1) and for all age subgroups (Supplementary Table SA3–10). Recent studies, in fact, consider standard HRV analysis to be mathematically biased because of the nonlinear relationship between RR interval and HR, especially if patients differ in terms of their mean HR (101–103). To overcome this bias while retaining the physiological and clinical significance of HRV, HRV parameters were calculated relative to the mean RR value, i.e., fluctuations were normalized to the mean (101, 102, 104). This normalization is particularly important if HRV is compared in patients with a different mean HR or during interventions that result in a change in HR (6). This approach was developed to improve or completely remove the influence of HR, the strongest determinant that can influence HRV (1, 106, 115). Thus, the baseline values of HRVcorr parameters provided by the present study can be used in clinical practice as a benchmark during CC assessment and the rehabilitation period. In addition to determining whether a given value is within normal limits, the automatic HRVcorr calculator that recalculates standard HRV parameters and converts them into corrected parameters facilitates its clinical interpretation (Supplementary Appendix: Excel sheet) (106). Such a correction therefore allows for the exploration of accurate clinical values reflecting the actual physiological alterations related to the traumatic brain injury, which minimizes the risk of bias during clinical interpretation.

Although age was a significant predictor of the majority of HRVcorr parameters, multiple regression analysis showed that its contribution was small. The models accounted for only 1%–13% of the total variance in HRVcorr indices, the contribution of age to these models was quite limited (*β* ranged from −0.001 to −0.23), and its effect size was very small (f2 < 0.043). Therefore, there was virtually no reason to establish normative values for age, and normal limits for HRVcorr were calculated for the entire study group (Table 5). Gąsior et al. (106) found similar results. In their models, age accounted for at most 9% of the variance in HRVcorr, the role of age was limited (*β* varied between −0.29 and 0.13), and its effect size was small (f2 ≤ 0.091) (106). Like us, Gąsior et al. (106) did not establish normative values for age, and only HR was included in the correction. Moreover, the normative values presented in the above-mentioned study can be used as reference values for the assessment and clinical follow-up of sedentary male and female children aged between 6 and 13 years when assessing and monitoring CC. Thus, our study can be viewed as a complement to that of Gąsior et al. (106), since it provides normative values for participants aged between 14 and 21 years. It remains to be stated that our participants were male competitive contact sport athletes whereas, in the study by Gąsior et al. (106), the sample consisted of sedentary male and female children.

In recent years, there has been a growing movement in the scientific community to standardize the methodological approach to the HRV measurement, suggesting a need for detailed explanations of the techniques and equipment used to obtain reliable and reproducible results for (73, 88, 97, 116). In this study, we aimed to provide essential details that may help clinicians reproduce our measurements. Indeed, the restrictions to follow were imposed on participants before and during the experiment. A control questionnaire was used to validate compliance with these restrictions. To measure HRV, we chose the “Team2 Pro by POLAR”, a piece of equipment frequently used by sports scientists that enables the collection of data for several athletes at once. Thus, the ready availability of this equipment facilitates the assessment of HRV by clinicians and sport health professionals. Our experimental protocol, moreover, may provide support for sports teams looking to establish specific HRV values for each of their athletes. These measurements could be made during annual pre-season evaluations and serve as a reference for evaluating and monitoring athletes in the event of CC. For HRV analysis, we chose “Kubios HRV” Standard, a free software commonly used by the scientific community (99, 100) which is very easy to access and use, thus facilitating HRV analysis. However, interpretation of the analysis results calls for certain skills on the part of health care professionals.

### Strengths, limitations and future recommendations

To our knowledge, no studies have yet been performed to establish corrected normative values for short-term, resting-state HRV parameters specific to male athletes in contact sports. Contact sports are characterized by a high risk of CC, and our normative HRV values could help clinicians analyze accurate clinical values that reflect the actual physiological alterations related to head injury, thus minimizing the risk of bias in clinical interpretation. Nevertheless, our study has a number of limitations. For one thing, we did not collect respiratory rate or tidal volume data in parallel with the HRV measurement, which could influence HRV estimates. We took care, however, to ask participants to count their spontaneous breaths for one minute of time before measuring HRV in order to ensure that the spontaneous respiratory rate had no values below 8 breaths per minute (0.15 Hz) that should not be interpreted. That the study population consisted of male athletes only was a second limitation. Further studies are needed to establish normative HRV values specific to a female population.

## Conclusion

This study provides for the first time corrected normative values of short-term and resting-state HRV parameters in competitive contact sport male athletes aged between 14 and 21 years. These values were developed independently of the main determinants of HRV, in particular mean HR. The baseline values for HRV parameters provided herein can be used in clinical practice when assessing and monitoring CCs and can assist in decision making for safe return to play. The detailed explanation of the methods and materials used in this study could help clinicians and sports teams establish specific HRV baseline values for each of their athletes. The automatic HRV_corr_ calculator that converts standard HRV parameters into corrected parameters and determines whether a given value is within normal limits facilitates its clinical interpretation.

## Data Availability

The raw data supporting the conclusions of this article will be made available by the authors, without undue reservation.

## References

[1] Task Force of the European Society of Cardiology the North American Society of Pacing and Electrophysiology. Heart rate variability, standards of measurement, physiological interpretation, and clinical use. Circulation. (1996) 93:1043–65. 10.1161/01.CIR.93.5.1043 8598068

[2] Shaffer F McCraty R Zerr CL. A healthy heart is not a metronome: an integrative review of the heart’s anatomy and heart rate variability. Front Psychol. (2014) 5:1040. 10.3389/fpsyg.2014.01040 25324790 PMC4179748

[3] Malliani A Pagani M Lombardi F Cerutti S. Cardiovascular neural regulation explored in the frequency domain. Circulation. (1991) 84(2):482–92. 10.1161/01.cir.84.2.482 1860193

[4] Franchini KG Cowley AW Jr. Autonomic control of cardiac function. Primer on the autonomic nervous system. London: Elsevier Academic Press (2004). pp. 134–8. 10.1016/B978-012589762-4/50035-9

[5] Cannon WB. Bodily changes in pain, hunger, fear, and rage: An account of recent researches into the function of emotional excitement. New York, NY, United States: D. Appleton and company (1915). 10.1037/10013-000

[6] Billman GE Huikuri HV Sacha J Trimmel K. An introduction to heart rate variability: methodological considerations and clinical applications. Front Physiol. (2015) 6:55. 10.3389/fphys.2015.00055 25762937 PMC4340167

[7] Billman GE. The LF/HF ratio does not accurately measure cardiac sympatho-vagal balance. Front Physiol. (2013) 4:26. 10.3389/fphys.2013.00026 23431279 PMC3576706

[8] Vinik AI Maser RE Mitchell BD Freeman R. Diabetic autonomic neuropathy. Diabetes Care. (2003) 26(5):1553–79. 10.2337/diacare.26.5.1553 12716821

[9] Rosengård-Bärlund M Bernardi L Fagerudd J Mäntysaari M Björkesten CA Lindholm H Early autonomic dysfunction in type 1 diabetes: a reversible disorder? Diabetologia. (2009) 52(6):1164. 10.1007/s00125-009-1340-9 19340407

[10] Pagani M Lucini D. Autonomic dysregulation in essential hypertension: insight from heart rate and arterial pressure variability. Auton Neurosci. (2001) 90(1-2):76–82. 10.1016/S1566-0702(01)00270-3 11485295

[11] Maule S Rabbia F Perni V Tosello F Bisbocci D Mulatero P Prolonged QT interval and reduced heart rate variability in patients with uncomplicated essential hypertension. Hypertens Res. (2008) 31(11):2003–10. 10.1291/hypres.31.2003 19098371

[12] De Jong MMJ Randall DC. Heart rate variability analysis in the assessment of autonomic function in heart failure. J Cardiovasc Nurs. (2005) 20(3):186–95. 10.1097/00005082-200505000-00010 15870589

[13] Killavuori K Toivonen L Näveri H Leinonen H. Reversal of autonomic derangements by physical training in chronic heart failure assessed by heart rate variability. Eur Heart J. (1995) 16(4):490–5. 10.1093/oxfordjournals.eurheartj.a060941 7671894

[14] Skrapari I Tentolouris N Perrea D Bakoyiannis C Papazafiropoulou A Katsilambros N. Baroreflex sensitivity in obesity: relationship with cardiac autonomic nervous system activity. Obesity. (2007) 15(7):1685–93. 10.1038/oby.2007.201 17636086

[15] Thayer JF Yamamoto SS Brosschot JF. The relationship of autonomic imbalance, heart rate variability and cardiovascular disease risk factors. Int J Cardiol. (2010) 141(2):122–31. 10.1016/j.ijcard.2009.09.543 19910061

[16] Moss D Lgos L Shaffer F. Don’t add or miss a beat: a special issue on current evidence and current practice in heart rate variability biofeedback. Biofeedback (Online). (2013) 41(3):83. 10.5298/1081-5937-41.3.09

[17] Kim SW Jeon HR Kim JY Kim Y. Heart rate variability among children with acquired brain injury. Ann Rehabil Med. (2017) 41(6):951. 10.5535/arm.2017.41.6.951 29354571 PMC5773438

[18] Francis HM Fisher A Rushby JA McDonald S. Reduced heart rate variability in chronic severe traumatic brain injury: association with impaired emotional and social functioning, and potential for treatment using biofeedback. Neuropsychol Rehabil. (2016) 26(1):103–25. 10.1080/09602011.2014.1003246 25627984

[19] Baguley IJ Heriseanu RE Felmingham KL Cameron ID. Dysautonomia and heart rate variability following severe traumatic brain injury. Brain Inj. (2006) 20(4):437–44. 10.1080/02699050600664715 16716989

[20] Keren O Yupatov S Radai M Elad-Yarum R Faraggi D Abboud S Heart rate variability (HRV) of patients with traumatic brain injury (TBI) during the post-insult sub-acute period. Brain Inj. (2005) 19(8):605–11. 10.1080/02699050400024946 16175814

[21] Biswas AK Scott WA Sommerauer JF Luckett PM. Heart rate variability after acute traumatic brain injury in children. Crit Care Med. (2000) 28(12):3907–12. 10.1097/00003246-200012000-00030 11153634

[22] Su C-F Kuo TB Kuo J-S Lai H-Y Chen HI. Sympathetic and parasympathetic activities evaluated by heart-rate variability in head injury of various severities. Clin Neurophysiol. (2005) 116(6):1273–9. 10.1016/j.clinph.2005.01.010 15978489

[23] Hendén PL Söndergaard S Rydenhag B Reinsfelt B Ricksten S-E Åneman A. Can baroreflex sensitivity and heart rate variability predict late neurological outcome in patients with traumatic brain injury? J Neurosurg Anesthesiol. (2014) 26(1):50–9. 10.1097/ANA.0b013e3182a47b62 24064714

[24] Ryan ML Thorson CM Otero CA Vu T Proctor KG. Clinical applications of heart rate variability in the triage and assessment of traumatically injured patients. Anesthesiol Res Pract. (2011) 2011:416590. 10.1155/2011/416590 21350685 PMC3038414

[25] Mowery NT Norris PR Riordan W Jenkins JM Williams AE Morris JA Jr. Cardiac uncoupling and heart rate variability are associated with intracranial hypertension and mortality: a study of 145 trauma patients with continuous monitoring. J Trauma Acute Care Surg. (2008) 65(3):621–7. 10.1097/TA.0b013e3181837980 18784576

[26] Kahraman S Dutton RP Hu P Stansbury L Xiao Y Stein DM Heart rate and pulse pressure variability are associated with intractable intracranial hypertension after severe traumatic brain injury. J Neurosurg Anesthesiol. (2010) 22(4):296–302. 10.1097/ANA.0b013e3181e25fc3 20622688

[27] Rapenne T Moreau D Lenfant F Vernet M Boggio V Cottin Y Could heart rate variability predict outcome in patients with severe head injury? A pilot study. J Neurosurg Anesthesiol. (2001) 13(3):260–8. 10.1097/00008506-200107000-00016 11426105

[28] Goldstein B Kempski MH DeKing DB Cox C DeLong DJ Kelly MM Autonomic control of heart rate after brain injury in children. Crit Care Med. (1996) 24(2):234–40. 10.1097/00003246-199602000-00009 8605794

[29] Goldstein B Toweill D Lai S Sonnenthal K Kimberly B. Uncoupling of the autonomic and cardiovascular systems in acute brain injury. Am J Physiol Regul Integr Comp Physiol. (1998) 275(4):R1287–R92. 10.1152/ajpregu.1998.275.4.R1287 9756562

[30] Flatt AA Wilkerson GB Allen JR Keith CM Esco MR. Daily heart rate variability before and after concussion in an American college football player. Sports. (2019) 7(5):97. 10.3390/sports7050097 31035597 PMC6572754

[31] Purkayastha S Williams B Murphy M Lyng S Sabo T Bell KR. Reduced heart rate variability and lower cerebral blood flow associated with poor cognition during recovery following concussion. Auton Neurosci. (2019) 220:102548. 10.1016/j.autneu.2019.04.004 31331690

[32] Paniccia M Verweel L Thomas SG Taha T Keightley M Wilson KE Heart rate variability following youth concussion: how do autonomic regulation and concussion symptoms differ over time postinjury? BMJ Open Sport Exerc Med. (2018) 4(1):e000355. 10.1136/bmjsem-2018-000355 30305921 PMC6173244

[33] Bishop S Dech R Baker T Butz M Aravinthan K Neary JP. Parasympathetic baroreflexes and heart rate variability during acute stage of sport concussion recovery. Brain Inj. (2017) 31(2):247–59. 10.1080/02699052.2016.1226385 28045562

[34] Senthinathan A Mainwaring LM Hutchison M. Heart rate variability of athletes across concussion recovery milestones: a preliminary study. Clin J Sport Med. (2017) 27(3):288–95. 10.1097/JSM.0000000000000337 27379659

[35] Abaji JP Curnier D Moore RD Ellemberg D. Persisting effects of concussion on heart rate variability during physical exertion. J Neurotrauma. (2016) 33(9):811–7. 10.1089/neu.2015.3989 26159461

[36] Hilz MJ DeFina PA Anders S Koehn J Lang CJ Pauli E Frequency analysis unveils cardiac autonomic dysfunction after mild traumatic brain injury. J Neurotrauma. (2011) 28(9):1727–38. 10.1089/neu.2010.1497 21355816

[37] La Fountaine MF Heffernan KS Gossett JD Bauman WA De Meersman RE. Transient suppression of heart rate complexity in concussed athletes. Auton Neurosci. (2009) 148(1):101–3. 10.1016/j.autneu.2009.03.001 19303821

[38] Gall B Parkhouse W Goodman D. Heart rate variability of recently concussed athletes at rest and exercise. Med Sci Sports Exercise. (2004) 36:1269–74. 10.1249/01.mss.0000135787.73757.4d 15292731

[39] McCrory P Meeuwisse W Dvorak J Aubry M Bailes J Broglio S Consensus statement on concussion in sport-the 5(th) international conference on concussion in sport held in Berlin, October 2016. Br J Sports Med. (2017) 51(11):838–47. 10.1136/bjsports-2017-097699 28446457

[40] Lane JC Arciniegas DB. Post-traumatic headache. Curr Treat Options Neurol. (2002) 4(1):89–104. 10.1007/s11940-002-0007-3 11734106

[41] Cohen M Oksenberg A Snir D Stern M Groswasser Z. Temporally related changes of sleep complaints in traumatic brain injured patients. J Neurol Neurosurg Psychiatry. (1992) 55(4):313–5. 10.1136/jnnp.55.4.313 1583518 PMC489047

[42] Beetar JT Guilmette TJ Sparadeo FR. Sleep and pain complaints in symptomatic traumatic brain injury and neurologic populations. Arch Phys Med Rehabil. (1996) 77(12):1298–302. 10.1016/S0003-9993(96)90196-3 8976315

[43] Rao V Rollings P. Sleep disturbances following traumatic brain injury. Curr Treat Options Neurol. (2002) 4(1):77–87. 10.1007/s11940-002-0006-4 11734105

[44] Capruso DX Levin HS. Cognitive impairment following closed head injury. Neurol Clin. (1992) 10(4):879–93. 10.1016/S0733-8619(18)30185-3 1435662

[45] Hurley RA Taber KH. Emotional disturbances following traumatic brain injury. Curr Treat Options Neurol. (2002) 4(1):59–75. 10.1007/s11940-002-0005-5 11734104

[46] Silver JM Hales RE Yudofsky M. Neuropsychiatric aspects of traumatic brain injury. Essentials of neuropsychiatry and behavioral neurosciences. Washington, DC: American Psychiatric Publication (2010). p. 223–74.

[47] Giza CC Choe MC Barlow KM. Determining if rest is best after concussion. JAMA Neurol. (2018) 75(4):399–400. 10.1001/jamaneurol.2018.0006 29507932

[48] Wang Y Nelson LD LaRoche AA Pfaller AY Nencka AS Koch KM Cerebral blood flow alterations in acute sport-related concussion. J Neurotrauma. (2016) 33(13):1227–36. 10.1089/neu.2015.4072 26414315 PMC4931342

[49] Liao D Barnes RW Chambless LE Simpson RJ Jr Sorlie P Heiss G Age, race, and sex differences in autonomic cardiac function measured by spectral analysis of heart rate variability—the ARIC study. Am J Cardiol. (1995) 76(12):906–12. 10.1016/S0002-9149(99)80260-4 7484830

[50] Young FL Leicht AS. Short-term stability of resting heart rate variability: influence of position and gender. Appl Physiol Nutr Metabol. (2011) 36(2):210–8. 10.1139/h10-103 21609282

[51] Sharma VK Subramanian SK Arunachalam V Rajendran R. Heart rate variability in adolescents–normative data stratified by sex and physical activity. J Clin Diagn Res. (2015) 9(10):CC08. 10.7860/JCDR/2015/15373.6662 26557514 PMC4625233

[52] Gutin B Howe C Johnson MH Humphries MC Snieder H Barbeau P. Heart rate variability in adolescents: relations to physical activity, fitness, and adiposity. Med Sci Sports Exerc. (2005) 37(11):1856–63. 10.1249/01.mss.0000175867.98628.27 16286853

[53] Antelmi I De Paula RS Shinzato AR Peres CA Mansur AJ Grupi CJ. Influence of age, gender, body mass index, and functional capacity on heart rate variability in a cohort of subjects without heart disease. Am J Cardiol. (2004) 93(3):381–5. 10.1016/j.amjcard.2003.09.065 14759400

[54] Molfino A Fiorentini A Tubani L Martuscelli M Fanelli FR Laviano A. Body mass index is related to autonomic nervous system activity as measured by heart rate variability. Eur J Clin Nutr. (2009) 63(10):1263–5. 10.1038/ejcn.2009.35 19471292

[55] Brown TE Beightol LA Koh J Eckberg DL. Important influence of respiration on human RR interval power spectra is largely ignored. J Appl Physiol. (1993) 75(5):2310–7. 10.1152/jappl.1993.75.5.2310 8307890

[56] Buchheit M Al Haddad H Laursen P Ahmaidi S. Effect of body posture on postexercise parasympathetic reactivation in men. Exp Physiol. (2009) 94(7):795–804. 10.1113/expphysiol.2009.048041 19395660

[57] Huikuri HV Niemelä MJ Ojala S Rantala A Ikäheimo MJ Airaksinen K. Circadian rhythms of frequency domain measures of heart rate variability in healthy subjects and patients with coronary artery disease. Effects of arousal and upright posture. Circulation. (1994) 90(1):121–6. 10.1161/01.cir.90.1.121 8025987

[58] Toscani L Gangemi P Parigi A Silipo R Ragghianti P Sirabella E Human heart rate variability and sleep stages. Ital J Neurol Sci. (1996) 17(6):437–9. 10.1007/bf01997720 8978452

[59] Burton A Rahman K Kadota Y Lloyd A Vollmer-Conna U. Reduced heart rate variability predicts poor sleep quality in a case–control study of chronic fatigue syndrome. Exp Brain Res. (2010) 204(1):71–8. 10.1007/s00221-010-2296-1 20502886

[60] McCraty R Atkinson M Tiller WA Rein G Watkins AD. The effects of emotions on short-term power spectrum analysis of heart rate variability. Am J Cardiol. (1995) 76(14):1089–93. 10.1016/S0002-9149(99)80309-9 7484873

[61] Gouvernement of Canada. Concussion in Sport. (2020). Available from: https://www.canada.ca/fr/sante-publique/services/maladies/commotions-cerebrales-signes-symptomes/commotions-cerebrales-sport-infographie.html

[62] Public Health Agency of Canada. Injury in Review, 2020 Edition: Spotlight on Traumatic Brain Injuries Across the Life Course. (2020). Available from: https://www.canada.ca/en/public-health/services/injury-prevention/canadian-hospitals-injury-reporting-prevention-program/injury-reports/2020-spotlight-traumatic-brain-injuries-life-course.html 10.24095/hpcdp.40.9.05 PMC7534559 32909940

[63] Marar M McIlvain NM Fields SK Comstock RD. Epidemiology of concussions among United States high school athletes in 20 sports. Am J Sports Med. (2012) 40(4):747–55. 10.1177/0363546511435626 22287642

[64] Delaney JS Lacroix VJ Leclerc S Johnston KM. Concussions among university football and soccer players. Clin J Sport Med. (2002) 12(6):331–8. 10.1097/00042752-200211000-00003 12466687

[65] Thomas S Reading J Shephard RJ. Revision of the physical activity readiness questionnaire (PAR-Q). Can J Sport Sci. (1992) 17(4):338–45. PMID: 1330274 1330274

[66] Hautala A Tulppo MP Mäkikallio TH Laukkanen R Nissilä S Huikuri HV. Changes in cardiac autonomic regulation after prolonged maximal exercise. Clin Physiol. (2001) 21(2):238–45. 10.1046/j.1365-2281.2001.00309.x 11318832

[67] Mourot L Bouhaddi M Tordi N Rouillon J-D Regnard J. Short-and long-term effects of a single bout of exercise on heart rate variability: comparison between constant and interval training exercises. Eur J Appl Physiol. (2004) 92(4-5):508–17. 10.1007/s00421-004-1119-0 15461995

[68] Quintana DS McGregor IS Guastella AJ Malhi GS Kemp AH. A meta-analysis on the impact of alcohol dependence on short-term resting-state heart rate variability: implications for cardiovascular risk. Alcohol Clin Exp Res. (2013) 37(Suppl 1):E23–9. 10.1111/j.1530-0277.2012.01913.x 22834996

[69] Quintana DS Guastella AJ McGregor IS Hickie IB Kemp AH. Moderate alcohol intake is related to increased heart rate variability in young adults: implications for health and well-being. Psychophysiology. (2013) 50(12):1202–8. 10.1111/psyp.12134 23941125

[70] Hayano J Yamada M Sakakibara Y Fujinami T Yokoyama K Watanabe Y Short-and long-term effects of cigarette smoking on heart rate variability. Am J Cardiol. (1990) 65(1):84–8. 10.1016/0002-9149(90)90030-5 2294686

[71] D’Alessandro A Boeckelmann I Hammwhöner M Goette A. Nicotine, cigarette smoking and cardiac arrhythmia: an overview. Eur J Prev Cardiol. (2012) 19(3):297–305. 10.1177/1741826711411738 22779085

[72] Mehta A Jain A Mehta M Billie M. Caffeine and cardiac arrhythmias. An experimental study in dogs with review of literature. Acta Cardiol. (1997) 52(3):273–83. PMID: 9217918 9217918

[73] Catai AM Pastre CM de Godoy MF da Silva E de Medeiros Takahashi AC Vanderlei LCM. Heart rate variability: are you using it properly? Standardisation checklist of procedures. Braz J Phys Ther. (2020) 24(2):91–102. 10.1016/j.bjpt.2019.02.006 30852243 PMC7082649

[74] Lu C-L Zou X Orr WC Chen J. Postprandial changes of sympathovagal balance measured by heart rate variability. Dig Dis Sci. (1999) 44(4):857–61. 10.1023/A:1026698800742 10219849

[75] Fagius J Berne C. Increase in muscle nerve sympathetic activity in humans after food intake. Clin Sci. (1994) 86(2):159–67. 10.1042/cs0860159 8143426

[76] Massin MM Maeyns K Withofs N Ravet F Gérard P. Circadian rhythm of heart rate and heart rate variability. Arch Dis Child. (2000) 83(2):179–82. 10.1136/adc.83.2.179 10906034 PMC1718415

[77] Yamamoto S Iwamoto M Inoue M Harada N. Evaluation of the effect of heat exposure on the autonomic nervous system by heart rate variability and urinary catecholamines. J Occup Health. (2007) 49(3):199–204. 10.1539/joh.49.199 17575400

[78] Fagius J Karhuvaara S. Sympathetic activity and blood pressure increases with bladder distension in humans. Hypertension. (1989) 14(5):511–7. 10.1161/01.hyp.14.5.511 2807512

[79] Ben-Dror I Weissman A Leurer M Eldor-Itskovitz J Lowenstein L. Alterations of heart rate variability in women with overactive bladder syndrome. Int Urogynecol J. (2012) 23(8):1081–6. 10.1007/s00192-012-1738-7 22491716

[80] Mehnert U Knapp PA Mueller N Reitz A Schurch B. Heart rate variability: an objective measure of autonomic activity and bladder sensations during urodynamics. Neurourol Urodyn. (2009) 28(4):313–9. 10.1002/nau.20641 19058189

[81] May M Jordan J. The osmopressor response to water drinking. Am J Physiol-Regul Integr Comp Physiol. (2011) 300(1):R40–6. 10.1152/ajpregu.00544.2010 21048076

[82] McHugh J Keller NR Appalsamy M Thomas SA Raj SR Diedrich A Portal osmopressor mechanism linked to transient receptor potential vanilloid 4 and blood pressure control. Hypertension. (2010) 55(6):1438–43. 10.1161/hypertensionaha.110.151860 20385965 PMC2965336

[83] Jordan J Shannon JR Black BK Ali Y Farley M Costa F The pressor response to water drinking in humans: a sympathetic reflex? Circulation. (2000) 101(5):504–9. 10.1161/01.cir.101.5.504 10662747

[84] Saboul D Pialoux V Hautier C. The breathing effect of the LF/HF ratio in the heart rate variability measurements of athletes. Eur J Sport Sci. (2014) 14(sup1):S282–8. 10.1080/17461391.2012.691116 24444219

[85] Rennie KL Hemingway H Kumari M Brunner E Malik M Marmot M. Effects of moderate and vigorous physical activity on heart rate variability in a British study of civil servants. Am J Epidemiol. (2003) 158(2):135–43. 10.1093/aje/kwg120 12851226

[86] Tuomainen P Peuhkurinen K Kettunen R Rauramaa R. Regular physical exercise, heart rate variability and turbulence in a 6-year randomized controlled trial in middle-aged men: the DNASCO study. Life Sci. (2005) 77(21):2723–34. 10.1016/j.lfs.2005.05.023 15978638

[87] Vanderlei LCM Pastre CM Hoshi RA Carvalho T Godoy M. Basic notions of heart rate variability and its clinical applicability. Braz J Cardiovasc Surg. (2009) 24(2):205–17. 10.1590/S0102-76382009000200018 19768301

[88] Shaffer F Ginsberg J. An overview of heart rate variability metrics and norms. Front Public Health. (2017) 5:258. 10.3389/fpubh.2017.00258 29034226 PMC5624990

[89] Hartikainen JE Tahvanainen KU Kuusela TA. Short-term measurement of heart rate variability. In: Malik M, editor. Clinical guide to cardiac autonomic tests. Dordrecht, Netherlands: Springer. (1998). p. 149–76. 10.1007/978-94-017-1057-2_6

[90] Angelone A Coulter NA Jr. Respiratory sinus arrhythmia: a frequency dependent phenomenon. J Appl Physiol. (1964) 19(3):479–82. 10.1152/jappl.1964.19.3.479 14173545

[91] Hirsch JA Bishop B. Respiratory sinus arrhythmia in humans: how breathing pattern modulates heart rate. Am J Physiol Heart Circ Physiol. (1981) 241(4):H620–9. 10.1152/ajpheart.1981.241.4.H620 7315987

[92] Perini R Orizio C Milesi S Biancardi L Baselli G Veicsteinas A. Body position affects the power spectrum of heart rate variability during dynamic exercise. Eur J Appl Physiol Occup Physiol. (1993) 66(3):207–13. 10.1007/BF00235095 8477675

[93] Martinmäki K Kinnunen H. RR interval measurement and heart rate variability in polar products. In: Polar R&D physiological research polar electro Oy Kempele. Finland (2011).

[94] Schonfelder M Hinterseher G Peter P Spitzenpfeil P. Scientific comparison of different online heart rate monitoring systems. Int J Telemed Appl. (2011) 2011:631848. 10.1155/2011/631848 21760780 PMC3134140

[95] Achten J Jeukendrup AE. Heart rate monitoring. Sports Med. (2003) 33(7):517–38. 10.2165/00007256-200333070-00004 12762827

[96] Akalan C Robergs RA Kravitz L. Prediction of VO2max from an individualized submaximal cycle ergometer protocol. J Exerc Physiol Online. (2008) 11(2):1–17.

[97] Quintana D Alvares GA Heathers J. Guidelines for Reporting Articles on Psychiatry and Heart rate variability (GRAPH): recommendations to advance research communication. Transl Psychiatry. (2016) 6(5):e803-e. 10.1038/tp.2016.73 27163204 PMC5070064

[98] Pöyhönen M Syväoja S Hartikainen J Ruokonen E Takala J. The effect of carbon dioxide, respiratory rate and tidal volume on human heart rate variability. Acta Anaesthesiol Scand. (2004) 48(1):93–101. 10.1111/j.1399-6576.2004.00272.x 14674979

[99] Tarvainen MP Lipponen J Niskanen J-P Ranta-aho PO. Kubios HRV (ver. 3.3.0). User’s Guide (2019). Available from: https://www.kubios.com/support/

[100] Tarvainen MP Niskanen J-P Lipponen JA Ranta-Aho PO Karjalainen PA. Kubios HRV–heart rate variability analysis software. Comput Methods Programs Biomed. (2014) 113(1):210–20. 10.1016/j.cmpb.2013.07.024 24054542

[101] Sacha J Pluta W. Alterations of an average heart rate change heart rate variability due to mathematical reasons. Int J Cardiol. (2008) 128(3):444–7. 10.1016/j.ijcard.2007.06.047 17689709

[102] Sacha J Pluta W Witosa A. Which heart rate is more variable: a slow or a fast one?–it depends on the method of heart rate variability analysis. Folia Cardiol. (2005) 12(suppl D):1–4.

[103] Sacha J Pluta W. Different methods of heart rate variability analysis reveal different correlations of heart rate variability spectrum with average heart rate. J Electrocardiol. (2005) 38(1):47–53. 10.1016/j.jelectrocard.2004.09.015 15660347

[104] Sacha J Sobon J Sacha K Barabach S. Heart rate impact on the reproducibility of heart rate variability analysis. Int J Cardiol. (2013) 168(4):4257–9. 10.1016/j.ijcard.2013.04.160 23680595

[105] Lee C-H Lee J-H Son J-W Kim U Park J-S Lee J Normative values of short-term heart rate variability parameters in Koreans and their clinical value for the prediction of mortality. Heart Lung Circ. (2018) 27(5):576–87. 10.1016/j.hlc.2017.04.009 28592377

[106] Gąsior JS Sacha J Pawłowski M Zieliński J Jeleń PJ Tomik A Normative values for heart rate variability parameters in school-aged children: simple approach considering differences in average heart rate. Front Physiol. (2018) 9:1495. 10.3389/fphys.2018.01495 30405445 PMC6207594

[107] Dantas EM Kemp AH Andreão RV da Silva VJD Brunoni AR Hoshi RA Reference values for short-term resting-state heart rate variability in healthy adults: results from the Brazilian longitudinal study of adult health—eLSA-brasil study. Psychophysiology. (2018) 55(6):e13052. 10.1111/psyp.13052 29292837

[108] Jarrin DC McGrath JJ Poirier P Séguin L Tremblay RE Montplaisir JY Short-term heart rate variability in a population-based sample of 10-year-old children. Pediatr Cardiol. (2015) 36(1):41–8. 10.1007/s00246-014-0962-y 25056158 PMC4457514

[109] Seppälä S Laitinen T Tarvainen MP Tompuri T Veijalainen A Savonen K Normal Values for heart rate variability parameters in children 6–8 years of age: the PANIC study. Clin Physiol Funct Imaging. (2014) 34(4):290–6. 10.1111/cpf.12096 24666688

[110] Michels N Clays E De Buyzere M Huybrechts I Marild S Vanaelst B Determinants and reference values of short-term heart rate variability in children. Eur J Appl Physiol. (2013) 113(6):1477–88. 10.1007/s00421-012-2572-9 23269492

[111] Nunan D Sandercock GR Brodie DA. A quantitative systematic review of normal values for short-term heart rate variability in healthy adults. Pacing Clin Electrophysiol. (2010) 33(11):1407–17. 10.1111/j.1540-8159.2010.02841.x 20663071

[112] Draghici AE Taylor JA. The physiological basis and measurement of heart rate variability in humans. J Physiol Anthropol. (2016) 35(1):22. 10.1186/s40101-016-0113-7 27680542 PMC5039876

[113] Lahiri MK Kannankeril PJ Goldberger JJ. Assessment of autonomic function in cardiovascular disease: physiological basis and prognostic implications. J Am Coll Cardiol. (2008) 51(18):1725–33. 10.1016/j.jacc.2008.01.038 18452777

[114] Dengel DR Bosch TA Burruss TP Fielding KA Engel BE Weir NL Body composition and bone mineral density of national football league players. J Strength Cond Res. (2014) 28(1):1–6. 10.1519/JSC.0000000000000299 24149760

[115] Tsuji H Venditti FJ Manders ES Evans JC Larson MG Feldman CL Determinants of heart rate variability. J Am Coll Cardiol. (1996) 28(6):1539–46. 10.1016/S0735-1097(96)00342-7 8917269

[116] Massaro S Pecchia L. Heart rate variability (HRV) analysis: a methodology for organizational neuroscience. Organ Res Methods. (2019) 22(1):354–93. 10.1177/1094428116681072

