# Supplementary material for: Normative values of resting heart rate variability in young male contact sport athletes: Reference values for the assessment and treatment of concussion
Source: Front Sports Act Living. 2023 Jan 9;4:730401. doi: 10.3389/fspor.2022.730401 (PMC9869270; doi:10.3389/fspor.2022.730401)
Supplement: Supplementary file 1 [file Datasheet1.pdf]

Normative values of resting heart rate variability in young male contact sport athletes: reference values for the assessment and treatment of concussion

Appendix

TABLE A1 | Determinants of standard frequency-domain HRV parameters calculated with autoregressive method.

Standard HRV parameter	Determinant	Parameters of multiple regression analysis					
		β	P	Partial corr.	Multiple R^2	F-test	P
VLF (ln)	Mean HR	-0,50	<0,001	-0,48	0,25	23,59	<0,001
	Age (ln)	-0,05	0,31	-0,05			
	BMI (ln)	0,001	0,92	0,001			
	n.mTIB	0,09	<0,05	0,09			
	WSP (ln)	-0,03	0,54	-0,03			
LF (ln)	Mean HR	-0,26	<0,001	-0,25	0,09	6,99	<0,001
	Age (ln)	0,001	0,93	0,001			
	BMI (ln)	-0,08	0,14	-0,08			
	n.mTIB	0,08	0,13	0,08			
	WSP (ln)	0,05	0,33	0,05			
HF (ln)	Mean HR	-0,78	<0,001	-0,75	0,58	99,10	<0,001
	Age (ln)	-0,16	<0,001	-0,13			
	BMI (ln)	0,12	<0,001	0,11			
	n.mTIB	0,02	0,63	0,02			
	WSP (ln)	-0,08	<0,03	-0,07			
TP1 (ln)	Mean HR	-0,67	<0,001	-0,64	0,43	53,92	<0,001
	Age (ln)	-0,11	<0,02	-0,10			
	BMI (ln)	0,05	0,21	0,05			
	n.mTIB	0,04	0,32	0,04			
	WSP (ln)	-0,03	0,50	-0,03			
TP2 (ln)	Mean HR	-0,67	<0,001	-0,64	0,43	53,72	<0,001
	Age (ln)	-0,11	<0,02	-0,10			
	BMI (ln)	0,06	0,18	0,05			
	n.mTIB	0,04	0,35	0,04			
	WSP (ln)	-0,03	0,49	-0,03			
LF/HF (ln)	Mean HR	0,65	<0,001	0,63	0,43	54,25	<0,001
	Age (ln)	0,18	<0,001	0,16			
	BMI (ln)	-0,21	<0,001	-0,19			
	n.mTIB	0,05	0,20	0,05			
	WSP (ln)	0,14	<0,001	0,13			
nLF	Mean HR	0,65	<0,001	0,63	0,43	54,52	<0,001
	Age (ln)	0,18	<0,001	0,16			
	BMI (ln)	-0,21	<0,001	-0,19			
	n.mTIB	0,05	0,25	0,05			
	WSP (ln)	0,15	<0,001	0,14			
nHF	Mean HR	-0,65	<0,001	-0,63	0,43	54,59	<0,001
	Age (ln)	-0,18	<0,001	-0,16			
	BMI (ln)	0,21	<0,001	0,19			
	n.mTIB	-0,05	0,24	-0,05			
	WSP (ln)	-0,15	<0,001	-0,13			

TABLE A2 | Determinants and Cohen's f^2 indexes for corrected frequency-domain HRV parameters calculated with autoregressive method.

Standard HRV parameter	Determinant	Parameters of multiple regression analysis						Cohen's f^2	
		β	P	Partial correlation	Multiple R^2	F-test	P	Local	Combined
Corr-VLF	Age (months)	-0,06	0,29	-0,06	0,015	1,37	0,244	0,003	<0,001
	BMI	0,02	0,69	0,02				0,001	
	n.mTIB	0,10	<0,05	0,10				0,010	
	WSP	-0,05	0,39	-0,04				0,002	
Corr-LF	Age (months)	0,01	0,88	0,01	0,016	1,44	0,219	0,001	<0,001
	BMI	-0,06	0,31	-0,05				0,003	
	n.mTIB	0,09	0,08	0,09				0,008	
	WSP	0,06	0,27	0,06				0,003	
Corr-HF	Age (months)	-0,23	<0,001	-0,20	0,062	6,01	<0,001	0,040	<0,01
	BMI	0,16	<0,01	0,14				0,021	
	n.mTIB	0,02	0,70	0,02				0,001	
	WSP	-0,09	0,11	-0,08				0,007	
Corr-TP1	Age (months)	-0,12	<0,05	-0,10	0,015	1,35	0,251	0,011	<0,001
	BMI	0,05	0,37	0,05				0,002	
	n.mTIB	0,07	0,22	0,06				0,004	
	WSP	-0,01	0,83	-0,01				0,001	
Corr-TP2	Age (months)	-0,12	<0,05	-0,10	0,015	1,34	0,253	0,011	<0,001
	BMI	0,05	0,36	0,05				0,002	
	n.mTIB	0,06	0,24	0,06				0,004	
	WSP	-0,01	0,85	-0,01				0,001	
Corr-LF/HF	Age (months)	0,18	<0,001	0,16	0,087	8,66	<0,001	0,025	<0,01
	BMI	-0,19	<0,001	-0,17				0,031	
	n.mTIB	0,04	0,48	0,04				0,001	
	WSP	0,18	<0,001	0,17				0,029	
Corr-nLF	Age (months)	0,19	<0,001	0,16	0,093	9,33	<0,001	0,027	<0,01
	BMI	-0,22	<0,001	-0,21				0,044	
	n.mTIB	0,05	0,28	0,05				0,003	
	WSP	0,17	<0,001	0,15				0,025	
Corr-nHF	Age (months)	-0,23	<0,001	-0,20	0,130	13,61	<0,001	0,043	<0,05
	BMI	0,27	<0,001	0,25				0,065	
	n.mTIB	-0,05	0,36	-0,05				0,002	
	WSP	-0,19	<0,001	-0,18				0,033	

Corrected HRV rameters were calculated as follows: corr-SDNN, SDNN/mRRˆ1.2; corr-RMSSD, RMSSD/mRRˆ2.0; corrpNN50, pNN50/mRRˆ4.35; for FFT: corr-VLF, VLF/mRRˆ2.1; corr-LF, LF/mRRˆ1.55; corr-HF, HF/mRRˆ4.5; corr-TP1, TP1/mRRˆ3.1; corr-TP2, TP2/mRRˆ3.1; corr-LF/HF, LF/HF mRRˆ3.1; corr-nLF, nLF mRRˆ1.6; and corr-nHF, nHF/mRRˆ1.33. for AR: corr-VLF, VLF/mRRˆ2.0; corr-LF, LF/mRRˆ1.45; corr-HF, HF/mRRˆ4.65; corr-TP1, TP1/mRRˆ3.15; corr-TP2, TP2/mRRˆ3.25; corr-LF/HF, LF/HF mRRˆ3.4; corr-nLF, nLF mRRˆ1.85; and corr-nHF, nHF/mRRˆ1.4.

TABLE A3 | Determinants of standard time and frequency-domain HRV parameters calculated with fast-Fourier transform in athletes aged 14-15 years.

Standard HRV parameter	Determinant	Parameters of multiple regression analysis					
		β	P	Partial corr.	Multiple R^2	F-test	P
SDNN (ln)	Mean HR	-0,49	<0,001	-0,47	0,26	5,69	<0,001
	Age (ln)	-0,19	0,07	-0,18			
	BMI (ln)	-0,04	0,72	-0,03			
	n.mTIB	-0,04	0,70	-0,04			
	WSP (ln)	-0,14	0,14	-0,14			
RMSSD (ln)	Mean HR	-0,68	<0,001	-0,66	0,48	15,12	<0,001
	Age (ln)	-0,20	<0,02	-0,19			
	BMI (ln)	0,02	0,78	0,02			
	n.mTIB	-0,05	0,55	-0,05			
	WSP (ln)	-0,16	<0,05	-0,16			
pNN50 (ln)	Mean HR	-0,68	<0,001	-0,66	0,48	15,07	<0,001
	Age (ln)	-0,19	<0,03	-0,18			
	BMI (ln)	0,02	0,85	0,02			
	n.mTIB	-0,04	0,60	-0,04			
	WSP (ln)	-0,16	<0,05	-0,16			
VLF (ln)	Mean HR	-0,32	<0,001	-0,31	0,12	2,32	<0,05
	Age (ln)	0,07	0,51	0,07			
	BMI (ln)	-0,07	0,54	-0,06			
	n.mTIB	0,01	0,92	0,01			
	WSP (ln)	0,10	0,33	0,10			
LF (ln)	Mean HR	-0,24	<0,03	-0,23	0,08	1,53	0,19
	Age (ln)	-0,08	0,48	-0,07			
	BMI (ln)	-0,17	0,13	-0,16			
	n.mTIB	-0,03	0,77	-0,03			
	WSP (ln)	-0,07	0,51	-0,07			
HF (ln)	Mean HR	-0,66	<0,001	-0,63	0,47	14,51	<0,001
	Age (ln)	-0,20	<0,02	-0,19			
	BMI (ln)	0,07	0,41	0,07			
	n.mTIB	-0,08	0,36	-0,07			
	WSP (ln)	-0,14	0,09	-0,14			
TP1 (ln)	Mean HR	-0,58	<0,001	-0,56	0,35	9,08	<0,001
	Age (ln)	-0,18	<0,06	-0,17			
	BMI (ln)	-0,01	0,91	-0,01			
	n.mTIB	-0,07	0,44	-0,07			
	WSP (ln)	-0,13	0,14	-0,13			
TP2 (ln)	Mean HR	-0,58	<0,001	-0,56	0,36	9,14	<0,001
	Age (ln)	-0,19	<0,05	-0,17			
	BMI (ln)	-0,01	0,88	-0,01			
	n.mTIB	-0,07	0,43	-0,07			
	WSP (ln)	-0,14	0,12	-0,14			
LF/HF (ln)	Mean HR	0,57	<0,001	0,55	0,41	11,45	<0,001
	Age (ln)	0,17	0,07	0,16			
	BMI (ln)	-0,22	<0,02	-0,20			
	n.mTIB	0,06	0,47	0,06			
	WSP (ln)	0,11	0,21	0,11			
nLF	Mean HR	0,57	<0,001	0,55	0,40	11,15	<0,001
	Age (ln)	0,17	0,07	0,15			
	BMI (ln)	-0,21	<0,02	-0,20			
	n.mTIB	0,06	0,52	0,05			
	WSP (ln)	0,11	0,21	0,11			
nHF	Mean HR	-0,57	<0,001	-0,55	0,40	10,95	<0,001
	Age (ln)	-0,17	0,07	-0,15			
	BMI (ln)	0,21	<0,02	0,20			
	n.mTIB	-0,06	0,53	-0,05			
	WSP (ln)	-0,11	0,19	-0,11			

TABLE A4 | Determinants of standard frequency-domain HRV parameters calculated with autoregressive method in athletes aged 14-15 years.

Standard HRV parameter	Determinant	Parameters of multiple regression analysis					
		β	P	Partial corr.	Multiple R^2	F-test	P
VLF (ln)	Mean HR	-0,47	<0,001	-0,45	0,22	4,55	<0,001
	Age (ln)	-0,01	0,91	-0,01			
	BMI (ln)	-0,07	0,50	-0,07			
	n.mTIB	-0,05	0,65	-0,04			
	WSP (ln)	-0,07	0,47	-0,07			
LF (ln)	Mean HR	-0,17	0,14	-0,16	0,06	0,99	0,428
	Age (ln)	-0,08	0,47	-0,08			
	BMI (ln)	-0,16	0,17	-0,15			
	n.mTIB	-0,02	0,84	-0,02			
	WSP (ln)	-0,03	0,78	-0,03			
HF (ln)	Mean HR	-0,68	<0,001	-0,65	0,49	15,74	<0,001
	Age (ln)	-0,20	<0,02	-0,19			
	BMI (ln)	0,08	0,31	0,08			
	n.mTIB	-0,05	0,50	-0,05			
	WSP (ln)	-0,14	0,08	-0,14			
TP1 (ln)	Mean HR	-0,60	<0,001	-0,58	0,37	9,85	<0,001
	Age (ln)	-0,17	0,07	-0,16			
	BMI (ln)	0,001	0,98	0,001			
	n.mTIB	-0,06	0,48	-0,06			
	WSP (ln)	-0,13	0,16	-0,12			
TP2 (ln)	Mean HR	-0,59	<0,001	-0,57	0,37	9,66	<0,001
	Age (ln)	-0,18	<0,05	-0,17			
	BMI (ln)	0,01	0,94	0,01			
	n.mTIB	-0,06	0,50	-0,06			
	WSP (ln)	-0,13	0,16	-0,12			
LF/HF (ln)	Mean HR	0,57	<0,001	0,55	0,38	10,24	<0,001
	Age (ln)	0,17	0,07	0,16			
	BMI (ln)	-0,19	<0,04	-0,18			
	n.mTIB	0,01	0,90	0,01			
	WSP (ln)	0,11	0,21	0,11			
nLF	Mean HR	0,57	<0,001	0,55	0,39	10,53	<0,001
	Age (ln)	0,15	0,12	0,14			
	BMI (ln)	-0,20	<0,03	-0,19			
	n.mTIB	0,03	0,71	0,03			
	WSP (ln)	0,12	0,18	0,12			
nHF	Mean HR	-0,57	<0,001	-0,55	0,39	10,49	<0,001
	Age (ln)	-0,15	0,12	-0,14			
	BMI (ln)	0,20	<0,03	0,19			
	n.mTIB	-0,03	0,71	-0,03			
	WSP (ln)	-0,12	0,17	-0,12			

TABLE A5 | Determinants of standard time and frequency-domain HRV parameters calculated with fast-Fourier transform in athletes aged 16-17 years.

Standard HRV parameter	Determinant	Parameters of multiple regression analysis					
		β	P	Partial corr.	Multiple R^2	F-test	P
SDNN (ln)	Mean HR	-0,50	<0,001	-0,48	0,33	10,42	<0,001
	Age (ln)	0,03	0,69	0,03			
	BMI (ln)	0,16	<0,05	0,16			
	n.mTIB	0,10	0,22	0,10			
	WSP (ln)	0,05	0,54	0,05			
RMSSD (ln)	Mean HR	-0,68	<0,001	-0,65	0,49	20,84	<0,001
	Age (ln)	-0,03	0,70	-0,03			
	BMI (ln)	0,16	<0,02	0,16			
	n.mTIB	0,05	0,48	0,05			
	WSP (ln)	-0,05	0,46	-0,05			
pNN50 (ln)	Mean HR	-0,70	<0,001	-0,67	0,53	24,03	<0,001
	Age (ln)	0,001	0,97	0,001			
	BMI (ln)	0,21	<0,001	0,20			
	n.mTIB	0,04	0,54	0,04			
	WSP (ln)	-0,11	0,12	-0,11			
VLF (ln)	Mean HR	-0,32	<0,001	-0,31	0,16	4,04	<0,01
	Age (ln)	0,14	0,13	0,13			
	BMI (ln)	0,05	0,55	0,05			
	n.mTIB	0,08	0,35	0,08			
	WSP (ln)	0,05	0,63	0,04			
LF (ln)	Mean HR	-0,30	<0,001	-0,29	0,17	4,27	<0,001
	Age (ln)	0,11	0,21	0,11			
	BMI (ln)	-0,04	0,68	-0,04			
	n.mTIB	0,08	0,40	0,07			
	WSP (ln)	0,15	0,13	0,14			
HF (ln)	Mean HR	-0,72	<0,001	-0,69	0,59	30,33	<0,001
	Age (ln)	0,01	0,83	0,01			
	BMI (ln)	0,24	<0,001	0,24			
	n.mTIB	0,03	0,64	0,03			
	WSP (ln)	-0,05	0,49	-0,04			
TP1 (ln)	Mean HR	-0,63	<0,001	-0,60	0,46	18,17	<0,001
	Age (ln)	0,07	0,37	0,06			
	BMI (ln)	0,16	<0,03	0,16			
	n.mTIB	0,06	0,44	0,06			
	WSP (ln)	0,03	0,71	0,03			
TP2 (ln)	Mean HR	-0,63	<0,001	-0,60	0,46	18,25	<0,001
	Age (ln)	0,06	0,40	0,06			
	BMI (ln)	0,16	<0,03	0,16			
	n.mTIB	0,06	0,44	0,06			
	WSP (ln)	0,03	0,74	0,02			
LF/HF (ln)	Mean HR	0,60	<0,001	0,57	0,45	17,26	<0,001
	Age (ln)	0,09	0,24	0,08			
	BMI (ln)	-0,33	<0,001	-0,32			
	n.mTIB	0,03	0,66	0,03			
	WSP (ln)	0,19	<0,02	0,18			
nLF	Mean HR	0,59	<0,001	0,57	0,44	17,12	<0,001
	Age (ln)	0,08	0,27	0,08			
	BMI (ln)	-0,33	<0,001	-0,32			
	n.mTIB	0,03	0,64	0,03			
	WSP (ln)	0,18	<0,02	0,17			
nHF	Mean HR	-0,59	<0,001	-0,57	0,44	16,96	<0,001
	Age (ln)	-0,08	0,27	-0,08			
	BMI (ln)	0,33	<0,001	0,32			
	n.mTIB	-0,04	0,63	-0,04			
	WSP (ln)	-0,18	<0,02	-0,17			

TABLE A6 | Determinants of standard frequency-domain HRV parameters calculated with autoregressive method in athletes aged 16-17 years.

Standard HRV parameter	Determinant	Parameters of multiple regression analysis					
		β	P	Partial corr.	Multiple R^2	F-test	P
VLF (ln)	Mean HR	-0,44	<0,001	-0,42	0,27	7,78	<0,001
	Age (ln)	0,16	0,06	0,16			
	BMI (ln)	0,05	0,53	0,05			
	n.mTIB	0,12	0,16	0,12			
	WSP (ln)	0,03	0,77	0,02			
LF (ln)	Mean HR	-0,23	<0,01	-0,22	0,15	3,77	<0,01
	Age (ln)	0,08	0,40	0,07			
	BMI (ln)	0,03	0,78	0,03			
	n.mTIB	0,11	0,23	0,11			
	WSP (ln)	0,19	0,05	0,18			
HF (ln)	Mean HR	-0,76	<0,001	-0,72	0,63	36,76	<0,001
	Age (ln)	0,01	0,87	0,01			
	BMI (ln)	0,23	<0,001	0,23			
	n.mTIB	0,04	0,46	0,04			
	WSP (ln)	-0,06	0,32	-0,06			
TP1 (ln)	Mean HR	-0,63	<0,001	-0,61	0,48	20,14	<0,001
	Age (ln)	0,05	0,48	0,05			
	BMI (ln)	0,18	<0,01	0,18			
	n.mTIB	0,07	0,31	0,07			
	WSP (ln)	0,03	0,66	0,03			
TP2 (ln)	Mean HR	-0,64	<0,001	-0,61	0,49	20,48	<0,001
	Age (ln)	0,04	0,57	0,04			
	BMI (ln)	0,19	<0,01	0,18			
	n.mTIB	0,07	0,33	0,07			
	WSP (ln)	0,03	0,64	0,03			
LF/HF (ln)	Mean HR	0,62	<0,001	0,60	0,42	15,47	<0,001
	Age (ln)	0,09	0,26	0,08			
	BMI (ln)	-0,20	<0,01	-0,20			
	n.mTIB	0,02	0,77	0,02			
	WSP (ln)	0,25	<0,001	0,23			
nLF	Mean HR	0,63	<0,001	0,60	0,43	16,09	<0,001
	Age (ln)	0,06	0,44	0,06			
	BMI (ln)	-0,23	<0,001	-0,22			
	n.mTIB	0,05	0,51	0,05			
	WSP (ln)	0,26	<0,001	0,24			
nHF	Mean HR	-0,63	<0,001	-0,60	0,43	16,08	<0,001
	Age (ln)	-0,06	0,43	-0,06			
	BMI (ln)	0,23	<0,001	0,23			
	n.mTIB	-0,05	0,51	-0,05			
	WSP (ln)	-0,25	<0,001	-0,23			

TABLE A7 | Determinants of standard time and frequency-domain HRV parameters calculated with fast-Fourier transform in athletes aged 18-19 years.

Standard HRV parameter	Determinant	Parameters of multiple regression analysis					
		β	P	Partial corr.	Multiple R^2	F-test	P
SDNN (ln)	Mean HR	-0,51	<0,001	-0,51	0,26	8,18	<0,001
	Age (ln)	-0,09	0,29	-0,09			
	BMI (ln)	0,01	0,97	0,001			
	n.mTIB	0,04	0,59	0,04			
	WSP (ln)	-0,01	0,88	-0,01			
RMSSD (ln)	Mean HR	-0,69	<0,001	-0,68	0,47	20,07	<0,001
	Age (ln)	-0,08	0,28	-0,07			
	BMI (ln)	0,04	0,54	0,04			
	n.mTIB	0,03	0,72	0,02			
	WSP (ln)	-0,02	0,81	-0,02			
pNN50 (ln)	Mean HR	-0,70	<0,001	-0,70	0,50	22,44	<0,001
	Age (ln)	-0,07	0,29	-0,07			
	BMI (ln)	0,08	0,26	0,07			
	n.mTIB	0,07	0,34	0,06			
	WSP (ln)	-0,05	0,43	-0,05			
VLF (ln)	Mean HR	-0,35	<0,001	-0,35	0,14	3,59	<0,01
	Age (ln)	-0,13	0,15	-0,13			
	BMI (ln)	0,02	0,84	0,02			
	n.mTIB	0,01	0,94	0,01			
	WSP (ln)	-0,01	0,94	-0,01			
LF (ln)	Mean HR	-0,37	<0,001	-0,37	0,16	4,25	<0,001
	Age (ln)	-0,06	0,50	-0,06			
	BMI (ln)	-0,09	0,29	-0,09			
	n.mTIB	0,06	0,50	0,06			
	WSP (ln)	-0,01	0,91	-0,01			
HF (ln)	Mean HR	-0,75	<0,001	-0,75	0,56	28,99	<0,001
	Age (ln)	-0,05	0,44	-0,05			
	BMI (ln)	0,05	0,41	0,05			
	n.mTIB	0,02	0,72	0,02			
	WSP (ln)	-0,08	0,24	-0,07			
TP1 (ln)	Mean HR	-0,65	<0,001	-0,64	0,42	16,51	<0,001
	Age (ln)	-0,04	0,54	-0,04			
	BMI (ln)	-0,01	0,84	-0,01			
	n.mTIB	0,04	0,62	0,03			
	WSP (ln)	-0,04	0,59	-0,04			
TP2 (ln)	Mean HR	-0,65	<0,001	-0,64	0,42	16,54	<0,001
	Age (ln)	-0,04	0,58	-0,04			
	BMI (ln)	-0,01	0,84	-0,01			
	n.mTIB	0,04	0,61	0,04			
	WSP (ln)	-0,04	0,56	-0,04			
LF/HF (ln)	Mean HR	0,60	<0,001	0,60	0,38	13,81	<0,001
	Age (ln)	0,01	1,00	0,001			
	BMI (ln)	-0,18	<0,02	-0,17			
	n.mTIB	0,04	0,63	0,04			
	WSP (ln)	0,09	0,23	0,09			
nLF	Mean HR	0,59	<0,001	0,59	0,37	13,30	<0,001
	Age (ln)	0,02	0,83	0,02			
	BMI (ln)	-0,19	<0,01	-0,19			
	n.mTIB	0,02	0,75	0,02			
	WSP (ln)	0,10	0,18	0,10			
nHF	Mean HR	-0,59	<0,001	-0,59	0,37	13,32	<0,001
	Age (ln)	-0,01	0,85	-0,01			
	BMI (ln)	0,19	<0,01	0,19			
	n.mTIB	-0,03	0,74	-0,03			
	WSP (ln)	-0,10	0,17	-0,10			

TABLE A8 | Determinants of standard frequency-domain HRV parameters calculated with autoregressive method in athletes aged 18-19 years.

Standard HRV parameter	Determinant	Parameters of multiple regression analysis					
		β	P	Partial corr.	Multiple R^2	F-test	P
VLF (ln)	Mean HR	-0,47	<0,001	-0,47	0,24	7,11	<0,001
	Age (ln)	-0,08	0,35	-0,08			
	BMI (ln)	0,01	0,90	0,01			
	n.mTIB	0,12	0,17	0,11			
	WSP (ln)	-0,05	0,57	-0,05			
LF (ln)	Mean HR	-0,31	<0,001	-0,31	0,12	3,05	<0,05
	Age (ln)	-0,08	0,36	-0,08			
	BMI (ln)	-0,07	0,43	-0,07			
	n.mTIB	0,08	0,38	0,08			
	WSP (ln)	-0,01	0,91	-0,01			
HF (ln)	Mean HR	-0,77	<0,001	-0,77	0,59	32,93	<0,001
	Age (ln)	-0,04	0,53	-0,04			
	BMI (ln)	0,05	0,37	0,05			
	n.mTIB	0,001	0,98	0,001			
	WSP (ln)	-0,08	0,17	-0,08			
TP1 (ln)	Mean HR	-0,65	<0,001	-0,65	0,43	16,99	<0,001
	Age (ln)	-0,05	0,47	-0,05			
	BMI (ln)	0,001	0,98	0,001			
	n.mTIB	0,03	0,65	0,03			
	WSP (ln)	-0,04	0,56	-0,04			
TP2 (ln)	Mean HR	-0,65	<0,001	-0,65	0,43	17,11	<0,001
	Age (ln)	-0,05	0,49	-0,05			
	BMI (ln)	0,001	0,97	0,001			
	n.mTIB	0,03	0,67	0,03			
	WSP (ln)	-0,04	0,54	-0,04			
LF/HF (ln)	Mean HR	0,59	<0,001	0,59	0,36	13,03	<0,001
	Age (ln)	0,001	0,97	0,001			
	BMI (ln)	-0,12	0,11	-0,12			
	n.mTIB	0,07	0,34	0,07			
	WSP (ln)	0,14	0,07	0,14			
nLF	Mean HR	0,63	<0,001	0,63	0,42	16,57	<0,001
	Age (ln)	-0,03	0,71	-0,03			
	BMI (ln)	-0,16	<0,03	-0,16			
	n.mTIB	0,08	0,29	0,08			
	WSP (ln)	0,11	0,12	0,11			
nHF	Mean HR	-0,63	<0,001	-0,63	0,42	16,63	<0,001
	Age (ln)	0,03	0,72	0,03			
	BMI (ln)	0,16	<0,03	0,16			
	n.mTIB	-0,08	0,28	-0,08			
	WSP (ln)	-0,11	0,12	-0,11			

Normative values of resting heart rate variability in young male contact sport athletes: reference values for the assessment and treatment of concussion

TABLE A9 | Determinants of standard time and frequency-domain HRV parameters calculated with fast-Fourier transform in athletes aged 20-21 years.

Standard HRV parameter	Determinant	Parameters of multiple regression analysis					
		β	P	Partial corr.	Multiple R^2	F-test	P
SDNN (ln)	Mean HR	-0,61	<0,001	-0,59	0,39	5,16	<0,001
	Age (ln)	-0,03	0,81	-0,03			
	BMI (ln)	0,08	0,55	0,07			
	n.mTIB	0,17	0,18	0,17			
	WSP (ln)	-0,11	0,40	-0,10			
RMSSD (ln)	Mean HR	-0,77	<0,001	-0,74	0,61	13,00	<0,001
	Age (ln)	-0,03	0,79	-0,03			
	BMI (ln)	0,14	0,16	0,14			
	n.mTIB	0,14	0,18	0,13			
	WSP (ln)	-0,08	0,47	-0,07			
pNN50 (ln)	Mean HR	-0,75	<0,001	-0,72	0,58	11,11	<0,001
	Age (ln)	-0,04	0,67	-0,04			
	BMI (ln)	0,11	0,31	0,10			
	n.mTIB	0,17	0,11	0,17			
	WSP (ln)	-0,09	0,43	-0,08			
VLF (ln)	Mean HR	-0,49	<0,001	-0,46	0,28	3,23	<0,05
	Age (ln)	0,04	0,78	0,04			
	BMI (ln)	-0,01	0,93	-0,01			
	n.mTIB	0,20	0,14	0,20			
	WSP (ln)	-0,26	0,07	-0,25			
LF (ln)	Mean HR	-0,51	<0,001	-0,48	0,29	3,33	<0,05
	Age (ln)	-0,06	0,67	-0,06			
	BMI (ln)	-0,09	0,50	-0,09			
	n.mTIB	0,20	0,14	0,20			
	WSP (ln)	-0,21	0,14	-0,20			
HF (ln)	Mean HR	-0,85	<0,001	-0,82	0,71	19,89	<0,001
	Age (ln)	-0,02	0,81	-0,02			
	BMI (ln)	0,08	0,34	0,08			
	n.mTIB	0,13	0,13	0,13			
	WSP (ln)	-0,15	0,10	-0,14			
TP1 (ln)	Mean HR	-0,79	<0,001	-0,76	0,61	12,74	<0,001
	Age (ln)	-0,03	0,74	-0,03			
	BMI (ln)	0,04	0,67	0,04			
	n.mTIB	0,16	0,12	0,16			
	WSP (ln)	-0,17	0,11	-0,16			
TP2 (ln)	Mean HR	-0,79	<0,001	-0,76	0,61	12,67	<0,001
	Age (ln)	-0,04	0,73	-0,03			
	BMI (ln)	0,04	0,66	0,04			
	n.mTIB	0,16	0,12	0,15			
	WSP (ln)	-0,17	0,12	-0,16			
LF/HF (ln)	Mean HR	0,69	<0,001	0,66	0,52	8,92	<0,001
	Age (ln)	-0,02	0,84	-0,02			
	BMI (ln)	-0,19	0,10	-0,18			
	n.mTIB	0,001	0,97	0,001			
	WSP (ln)	0,02	0,88	0,02			
nLF	Mean HR	0,69	<0,001	0,66	0,52	8,96	<0,001
	Age (ln)	-0,03	0,75	-0,03			
	BMI (ln)	-0,17	0,13	-0,17			
	n.mTIB	-0,02	0,83	-0,02			
	WSP (ln)	0,02	0,84	0,02			
nHF	Mean HR	-0,69	<0,001	-0,66	0,52	8,77	<0,001
	Age (ln)	0,04	0,75	0,03			
	BMI (ln)	0,17	0,14	0,16			
	n.mTIB	0,02	0,85	0,02			
	WSP (ln)	-0,02	0,85	-0,02			

TABLE A10 | Determinants of standard frequency-domain HRV parameters calculated with autoregressive method in athletes aged 20-21 years.

Standard HRV parameter	Determinant	Parameters of multiple regression analysis					
		β	P	Partial corr.	Multiple R^2	F-test	P
VLF (ln)	Mean HR	-0,71	<0,001	-0,68	0,50	8,31	<0,001
	Age (ln)	-0,10	0,36	-0,10			
	BMI (ln)	-0,04	0,71	-0,04			
	n.mTIB	0,20	0,08	0,20			
	WSP (ln)	-0,15	0,21	-0,14			
LF (ln)	Mean HR	-0,29	<0,06	-0,28	0,12	1,17	0,343
	Age (ln)	-0,03	0,83	-0,03			
	BMI (ln)	-0,08	0,61	-0,08			
	n.mTIB	0,21	0,17	0,20			
	WSP (ln)	-0,10	0,51	-0,10			
HF (ln)	Mean HR	-0,83	<0,001	-0,80	0,69	18,58	<0,001
	Age (ln)	-0,01	0,91	-0,01			
	BMI (ln)	0,14	0,13	0,13			
	n.mTIB	0,11	0,23	0,10			
	WSP (ln)	-0,12	0,20	-0,11			
TP1 (ln)	Mean HR	-0,75	<0,001	-0,72	0,56	10,52	<0,001
	Age (ln)	-0,04	0,70	-0,04			
	BMI (ln)	0,11	0,31	0,11			
	n.mTIB	0,13	0,22	0,13			
	WSP (ln)	-0,13	0,24	-0,12			
TP2 (ln)	Mean HR	-0,75	<0,001	-0,71	0,55	10,05	<0,001
	Age (ln)	-0,04	0,73	-0,04			
	BMI (ln)	0,12	0,29	0,11			
	n.mTIB	0,13	0,24	0,12			
	WSP (ln)	-0,13	0,25	-0,12			
LF/HF (ln)	Mean HR	0,74	<0,001	0,71	0,58	11,19	<0,001
	Age (ln)	-0,07	0,50	-0,07			
	BMI (ln)	-0,10	0,34	-0,10			
	n.mTIB	-0,07	0,48	-0,07			
	WSP (ln)	0,05	0,68	0,04			
nLF	Mean HR	0,76	<0,001	0,73	0,61	12,73	<0,001
	Age (ln)	-0,03	0,77	-0,03			
	BMI (ln)	-0,20	0,06	-0,19			
	n.mTIB	0,01	0,91	0,01			
	WSP (ln)	0,07	0,53	0,06			
nHF	Mean HR	-0,76	<0,001	-0,73	0,61	12,70	<0,001
	Age (ln)	0,03	0,77	0,03			
	BMI (ln)	0,20	0,06	0,19			
	n.mTIB	-0,01	0,91	-0,01			
	WSP (ln)	-0,07	0,53	-0,06			
